# MHC Hammer reveals genetic and non-genetic HLA disruption in cancer evolution

**DOI:** 10.1038/s41588-024-01883-8

**Published:** 2024-10-02

**Authors:** Clare Puttick, Thomas P. Jones, Michelle M. Leung, Felipe Galvez-Cancino, Jiali Liu, Manuel Varas-Godoy, Andrew Rowan, Oriol Pich, Carlos Martinez-Ruiz, Robert Bentham, Krijn K. Dijkstra, James R. M. Black, Rachel Rosenthal, Nnennaya Kanu, Kevin Litchfield, Roberto Salgado, David A. Moore, Peter Van Loo, Mariam Jamal-Hanjani, Sergio A. Quezada, TRACERx Consortium, Heather Cheyne, Mohammed Khalil, Shirley Richardson, Tracey Cruickshank, Eric Lim, Hugo J. W. L. Aerts, Tom L. Kaufmann, Matthew R. Huska, Babu Naidu, Gareth A. Wilson, Rachel Rosenthal, Andrew Rowan, Chris Bailey, Claudia Lee, Emma Colliver, Katey S. S. Enfield, Mark S. Hill, Mihaela Angelova, Oriol Pich, Dhruva Biswas, Clare Puttick, Roberto Vendramin, Cian Murphy, Maria Zagorulya, Thomas P. Jones, Michelle M. Leung, Nicholas McGranahan, Carla Castignani, Elizabeth Larose Cadieux, Jeanette Kittel, Kerstin Haase, Kexin Koh, Rachel Scott, Gurdeep Matharu, Jacqui A. Shaw, Allan Hackshaw, Camilla Pilotti, Rachel Leslie, Anne-Marie Hacker, Sean Smith, Aoife Walker, Christopher Abbosh, Corentin Richard, Cristina Naceur-Lombardelli, Francisco Gimeno-Valiente, Krupa Thakkar, Mariana Werner Sunderland, Monica Sivakumar, Nnennaya Kanu, Ieva Usaite, Sadegh Saghafinia, Selvaraju Veeriah, Sharon Vanloo, Bushra Mussa, Michalina Magala, Elizabeth Keene, Emilia L. Lim, James R. sM Black, Maise Al Bakir, Ariana Huebner, Kristiana Grigoriadis, Takahiro Karasaki, Alexander M. Frankell, Crispin T. Hiley, Sophia Ward, Sian Harries, Olivia Lucas, David A. Moore, Nicolai J. Birkbak, Carlos Martínez-Ruiz, Kerstin Thol, Robert Bentham, Wing Kin Liu, Abigail Bunkum, Sonya Hessey, Martin D. Forster, Siow Ming Lee, Mariam Jamal-Hanjani, Despoina Karagianni, Sergio A. Quezada, Supreet Kaur Bola, Kevin Litchfield, Charles Swanton, John Le Quesne, Khalid AbdulJabbar, Catarina Veiga, Simone Zaccaria, Jonathan Tugwood, Caroline Dive, Zoltan Szallasi, Miklos Diossy, Teresa Marafioti, Elaine Borg, Mary Falzon, Reena Khiroya, Peter Van Loo, Karl S. Peggs, Gillian Price, Gary Royle, Charles-Antoine Collins-Fekete, Dionysis Papadatos-Pastos, James Wilson, Tanya Ahmad, Sarah Benafif, Judith Cave, Keith M. Kerr, Thomas B. K. Watkins, Roberto Salgado, Alexander James Procter, Asia Ahmed, Magali N. Taylor, Arjun Nair, David Lawrence, Davide Patrini, Colin R. Lindsay, Fiona H. Blackhall, Yvonne Summers, Matthew G. Krebs, Emma Nye, Richard Kevin Stone, Hanyun Zhang, Jerome Nicod, Alan Kirk, Mo Asif, Rocco Bilancia, Nikos Kostoulas, Jennifer Whiteley, Mathew Thomas, Akshay J. Patel, David Chuter, Mairead MacKenzie, Roland F. Schwarz, Andrew Kidd, Francesco Fraioli, Paul Ashford, Zoltan Kaplar, Jonas Demeulemeester, Claire Wilson, Michael J. Shackcloth, Sam M. Janes, Neal Navani, Ricky M. Thakrar, Angela Leek, Jack Davies Hodgkinson, Nicola Totton, Antonio Paiva-Correia, Stephan Beck, Miljana Tanic, Craig Dick, Lily Robinson, Peter Russell, Paulo De Sousa, Simon Jordan, Alexandra Rice, Hilgardt Raubenheimer, Harshil Bhayani, Lyn Ambrose, Anand Devaraj, Hemangi Chavan, Sofina Begum, Silviu I. Buderi, Daniel Kaniu, Mpho Malima, Sarah Booth, Nadia Fernandes, Pratibha Shah, Chiara Proli, Andrew G. Nicholson, Ekaterini Boleti, Madeleine Hewish, Kevin G. Blyth, Jason F. Lester, Anshuman Chaturvedi, Pedro Oliveira, Katherine D. Brown, Mathew Carter, Alastair Magness, Clare E. Weeden, Eva Grönroos, Jacki Goldman, Mickael Escudero, Philip Hobson, Stefan Boeing, Tamara Denner, Vittorio Barbè, Wei-Ting Lu, William Hill, Yutaka Naito, Zoe Ramsden, George Kassiotis, Imran Noorani, Anca Grapa, Aiman Alzetani, Yinyin Yuan, Xiaoxi Pan, Jack French, Kayleigh Gilbert, Angela Dwornik, Angeliki Karamani, Benny Chain, David R. Pearce, Felip Gálvez-Cancino, Georgia Stavrou, Gerasimos-Theodoros Mastrokalos, Helen L. Lowe, Ignacio Garcia Matos, James L. Reading, John A. Hartley, Kayalvizhi Selvaraju, Kezhong Chen, Leah Ensell, Mansi Shah, Maria Litovchenko, Piotr Pawlik, Samuel Gamble, Seng Kuong Anakin Ung, Victoria Spanswick, Yin Wu, Jayant K. Rane, Othman Al-Sawaf, Olga Chervova, Emilie Martinoni Hoogenboom, Fleur Monk, James W. Holding, Junaid Choudhary, Kunal Bhakhri, Pat Gorman, Robert C. M. Stephens, Maria Chiara Pisciella, Steve Bandula, Yien Ning Sophia Wong, Aya Osman, Mandeesh Sangha, Gerald Langman, Helen Shackleford, Madava Djearaman, Gary Middleton, Serena Chee, Patricia Georg, Amrita Bajaj, Apostolos Nakas, Azmina Sodha-Ramdeen, Mohamad Tufail, Molly Scotland, Rebecca Boyles, Sridhar Rathinam, Domenic Marrone, Sean Dulloo, Dean A. Fennell, Sarah Danson, Elaine Smith, Eustace Fontaine, Felice Granato, Juliette Novasio, Kendadai Rammohan, Leena Joseph, Paul Bishop, Rajesh Shah, Vijay Joshi, Philip Crosbie, Charles Swanton, Nicholas McGranahan

**Affiliations:** 1 Cancer Evolution and Genome Instability Laboratory, The Francis Crick Institute, London, UK; 2 Cancer Genome Evolution Research Group, Cancer Research UK Lung Cancer Centre of Excellence, University College London Cancer Institute, London, UK; 3 Cancer Research UK Lung Cancer Centre of Excellence, University College London Cancer Institute, London, UK; 4 Cancer Immunology Unit, Immune Regulation and Tumour Immunotherapy Laboratory, Research Department of Haematology, University College London Cancer Institute, London, UK; 5 Cancer Cell Biology Laboratory, Centro de Biología Celular y Biomedicina (CEBICEM), Facultad de Medicina y Ciencia, Universidad San Sebastián, Santiago, Chile; 6 Centro Ciencia & Vida, Fundación Ciencia & Vida, Santiago, Chile; 7 Department of Molecular Oncology and Immunology, The Netherlands Cancer Institute, Amsterdam, The Netherlands; 8 Oncode Institute, Utrecht, The Netherlands; 9 Tumour Immunogenomics and Immunosurveillance Laboratory, University College London Cancer Institute, London, UK; 10 Division of Research, Peter MacCallum Cancer Centre, Melbourne, Australia; 11 Department of Pathology, ZAS Hospitals, Antwerp, Belgium; 12 Department of Cellular Pathology, University College London Hospitals, London, UK; 13 Cancer Genomics Laboratory, The Francis Crick Institute, London, UK; 14 Department of Genetics, The University of Texas MD Anderson Cancer Center, Houston, TX USA; 15 Department of Genomic Medicine, The University of Texas MD Anderson Cancer Center, Houston, TX USA; 16 Cancer Metastasis Laboratory, University College London Cancer Institute, London, UK; 17 Department of Medical Oncology, University College London Hospitals, London, UK; 18 Aberdeen Royal Infirmary NHS Grampian, Aberdeen, UK; 19 Academic Division of Thoracic Surgery, Imperial College London, London, UK; 20 Royal Brompton and Harefield Hospitals, part of Guy’s and St Thomas’ NHS Foundation Trust, London, UK; 21 Artificial Intelligence in Medicine (AIM) Program, Mass General Brigham, Harvard Medical School, Boston, MA USA; 22 Department of Radiation Oncology, Brigham and Women’s Hospital, Dana-Farber Cancer Institute, Harvard Medical School, Boston, MA USA; 23 Radiology and Nuclear Medicine, CARIM & GROW, Maastricht University, Maastricht, The Netherlands; 24 Berlin Institute for Medical Systems Biology, Max Delbrück Center for Molecular Medicine in the Helmholtz Association (MDC), Berlin, Germany; 25 Berlin Institute for the Foundations of Learning and Data (BIFOLD), Berlin, Germany; 26 Bioinformatics and Systems Biology, Method Development and Research Infrastructure, Robert Koch Institute, Berlin, Germany; 27 Birmingham Acute Care Research Group, Institute of Inflammation and Ageing, University of Birmingham, Birmingham, UK; 28 Bill Lyons Informatics Centre, University College London Cancer Institute, London, UK; 29 Medical Genomics, University College London Cancer Institute, London, UK; 30 Cancer Research Centre, University of Leicester, Leicester, UK; 31 Cancer Research UK & UCL Cancer Trials Centre, London, UK; 32 Department of Thoracic Surgery, Respiratory Center, Toranomon Hospital, Tokyo, Japan; 33 Advanced Sequencing Facility, The Francis Crick, London, UK; 34 Computational Cancer Genomics Research Group, University College London Cancer Institute, London, UK; 35 University College London Hospitals, London, UK; 36 Department of Molecular Medicine, Aarhus University Hospital, Aarhus, Denmark; 37 Department of Clinical Medicine, Aarhus University, Aarhus, Denmark; 38 Bioinformatics Research Centre, Aarhus University, Aarhus, Denmark; 39 Immune Regulation and Tumour Immunotherapy Group, Cancer Immunology Unit, Research Department of Haematology, University College London Cancer Institute, London, UK; 40 Cancer Research UK Scotland Institute, Glasgow, UK; 41 Institute of Cancer Sciences, University of Glasgow, Glasgow, UK; 42 NHS Greater Glasgow and Clyde Pathology Department, Queen Elizabeth University Hospital, Glasgow, UK; 43 The Institute of Cancer Research, London, UK; 44 Centre for Medical Image Computing, Department of Medical Physics and Biomedical Engineering, London, UK; 45 CRUK Manchester Institute Cancer Biomarker Centre, University of Manchester, Manchester, UK; 46 Cancer Research UK Lung Cancer Centre of Excellence, University of Manchester, Manchester, UK; 47 Danish Cancer Institute, Copenhagen, Denmark; 48 Computational Health Informatics Program, Boston Children’s Hospital, Boston, MA USA; 49 Department of Bioinformatics, Semmelweis University, Budapest, Hungary; 50 Department of Physics of Complex Systems, ELTE Eötvös Loránd University, Budapest, Hungary; 51 Department of Haematology, University College London Hospitals, London, UK; 52 Cancer Immunology Unit, Research Department of Haematology, University College London Cancer Institute, London, UK; 53 Department of Medical Oncology, Aberdeen Royal Infirmary NHS Grampian, Aberdeen, UK; 54 University of Aberdeen, Aberdeen, UK; 55 Department of Medical Physics and Bioengineering, University College London Cancer Institute, London, UK; 56 The Whittington Hospital NHS Trust, London, UK; 57 Department of Oncology, University Hospital Southampton NHS Foundation Trust, Southampton, UK; 58 Department of Pathology, Aberdeen Royal Infirmary NHS Grampian, Aberdeen, UK; 59 Department of Pathology, Stanford University School of Medicine, Stanford, CA USA; 60 Department of Radiology, University College London Hospitals, London, UK; 61 UCL Respiratory, Department of Medicine, University College London, London, UK; 62 Department of Thoracic Surgery, University College London Hospital NHS Trust, London, UK; 63 Division of Cancer Sciences, The University of Manchester and The Christie NHS Foundation Trust, Manchester, UK; 64 Experimental Histopathology, The Francis Crick Institute, London, UK; 65 Garvan Institute of Medical Research, Darlinghurst, New South Wales Australia; 66 Genomics Science Technology Platform, The Francis Crick Institute, London, UK; 67 Golden Jubilee National Hospital, Clydebank, UK; 68 Guy’s and St Thomas’ NHS Foundation Trust, London, UK; 69 Independent Cancer Patient’s Voice, London, UK; 70 Institute for Computational Cancer Biology, Center for Integrated Oncology (CIO), Cancer Research Center Cologne Essen (CCCE), Faculty of Medicine and University Hospital Cologne, University of Cologne, Cologne, Germany; 71 Institute of Infection, Immunity & Inflammation, University of Glasgow, Glasgow, UK; 72 Institute of Nuclear Medicine, Division of Medicine, University College London, London, UK; 73 Institute of Structural and Molecular Biology, University College London, London, UK; 74 Integrated Radiology Department, North-Buda St. John’s Central Hospital, Budapest, Hungary; 75 Institute of Nuclear Medicine, University College London Hospitals, London, UK; 76 Integrative Cancer Genomics Laboratory, VIB Center for Cancer Biology, Leuven, Belgium; 77 VIB Center for AI & Computational Biology, Leuven, Belgium; 78 Leicester Medical School, University of Leicester, Leicester, UK; 79 Liverpool Heart and Chest Hospital, Liverpool, UK; 80 Lungs for Living Research Centre, UCL Respiratory, Department of Medicine, University College London, London, UK; 81 Department of Thoracic Medicine, University College London Hospitals, London, UK; 82 Manchester Cancer Research Centre Biobank, Manchester, UK; 83 Manchester University NHS Foundation Trust, Manchester, UK; 84 Experimental Oncology, Institute for Oncology and Radiology of Serbia, Belgrade, Serbia; 85 NHS Greater Glasgow and Clyde, Glasgow, UK; 86 Princess Alexandra Hospital, The Princess Alexandra Hospital NHS Trust, Harlow, UK; 87 National Heart and Lung Institute, Imperial College, London, UK; 88 Royal Free London NHS Foundation Trust, London, UK; 89 Royal Surrey Hospital, Royal Surrey Hospitals NHS Foundation Trust, Guildford, UK; 90 University of Surrey, Guildford, UK; 91 School of Cancer Sciences, University of Glasgow, Glasgow, UK; 92 Beatson Institute for Cancer Research, University of Glasgow, Glasgow, UK; 93 Queen Elizabeth University Hospital, Glasgow, UK; 94 Singleton Hospital, Swansea Bay University Health Board, Swansea, UK; 95 The Christie NHS Foundation Trust, Manchester, UK; 96 The Francis Crick Institute, London, UK; 97 Department of Infectious Disease, Faculty of Medicine, Imperial College London, London, UK; 98 Department of Neurosurgery, National Hospital for Neurology and Neurosurgery, London, UK; 99 The NIHR Southampton Biomedical Research Centre, University Hospital Southampton NHS Foundation Trust, Southampton, UK; 100 The University of Texas MD Anderson Cancer Center, Houston, TX USA; 101 University College London Cancer Institute, London, UK; 102 Department I of Internal Medicine, University Hospital of Cologne, Cologne, Germany; 103 University College London Department of Epidemiology and Health Care, London, UK; 104 National Cancer Centre, Singapore City, Singapore; 105 University Hospital Birmingham NHS Foundation Trust, Birmingham, UK; 106 Institute of Immunology and Immunotherapy, University of Birmingham, Birmingham, UK; 107 University Hospital Southampton NHS Foundation Trust, Southampton, UK; 108 University Hospitals of Leicester NHS Trust, Leicester, UK; 109 University of Leicester, Leicester, UK; 110 University of Sheffield, Sheffield, UK; 111 Sheffield Teaching Hospitals NHS Foundation Trust, Sheffield, UK; 112 Wythenshawe Hospital, Manchester University NHS Foundation Trust, Wythenshawe, UK; 113 Division of Infection, Immunity and Respiratory Medicine, University of Manchester, Manchester, UK; 114 Immune Regulation and Immune Interactions Group, Centre for Immuno-Oncology, Nuffield Department of Medicine, University of Oxford, Oxford, UK

**Keywords:** Breast cancer, Genome informatics, Lung cancer, Tumour immunology

## Abstract

Disruption of the class I human leukocyte antigen (HLA) molecules has important implications for immune evasion and tumor evolution. We developed major histocompatibility complex loss of heterozygosity (LOH), allele-specific mutation and measurement of expression and repression (MHC Hammer). We identified extensive variability in HLA allelic expression and pervasive HLA alternative splicing in normal lung and breast tissue. In lung TRACERx and lung and breast TCGA cohorts, 61% of lung adenocarcinoma (LUAD), 76% of lung squamous cell carcinoma (LUSC) and 35% of estrogen receptor-positive (ER+) cancers harbored class I HLA transcriptional repression, while HLA tumor-enriched alternative splicing occurred in 31%, 11% and 15% of LUAD, LUSC and ER+ cancers. Consistent with the importance of HLA dysfunction in tumor evolution, in LUADs, HLA LOH was associated with metastasis and LUAD primary tumor regions seeding a metastasis had a lower effective neoantigen burden than non-seeding regions. These data highlight the extent and importance of HLA transcriptomic disruption, including repression and alternative splicing in cancer evolution.

## Abstract (web summary)

Major histocompatibility complex (MHC) loss of heterozygosity, allele-specific mutation and measurement of expression and repression (MHC Hammer) detects disruption to human leukocyte antigens due to mutations, loss of heterogeneity, altered gene expression or alternative splicing. Applied to lung and breast cancer datasets, the tool shows that these aberrations are common across cancer and can have clinical implications.

## Main

Emerging data have highlighted the importance of considering cancer evolution in the context of a predatory immune microenvironment^1–3^. Key mediators of the cytotoxic T cell response in cancer are neoantigens, cancer-cell-specific alterations resulting in mutant peptides capable of eliciting a T cell-mediated, human leukocyte antigen (HLA)-restricted immune response. A mutation can only result in a neoantigen if the associated mutant peptide is presented on HLA molecules to the T cell receptor. Therefore, disruption of HLA molecules has important implications for immune evasion.

Disruption to antigen-presenting machinery occurs across many cancer types^4–7^. Our previous work has revealed that HLA loss of heterozygosity (LOH), whereby one allele is somatically lost, occurs in 40% of non-small cell lung cancer (NSCLC) primary tumors^7^. A pan-cancer study has suggested that transcriptomic downregulation of HLA genes occurs frequently^4^. However, subtle transcriptomic alterations, such as alternative splicing events and allele-specific repression, have been poorly studied in cancer. Alternative splicing, which has been reported in non-cancer tissue and cancer cell lines, can result in a non-functional HLA molecule or, in the case of exon 5 skipping, soluble isoforms of the HLA molecule^8–13^. Furthermore, understanding HLA expression in tumor-adjacent normal tissue is of critical importance when attributing any change in HLA expression as a tumor-specific phenomenon.

**Extended Data Fig. 1:**
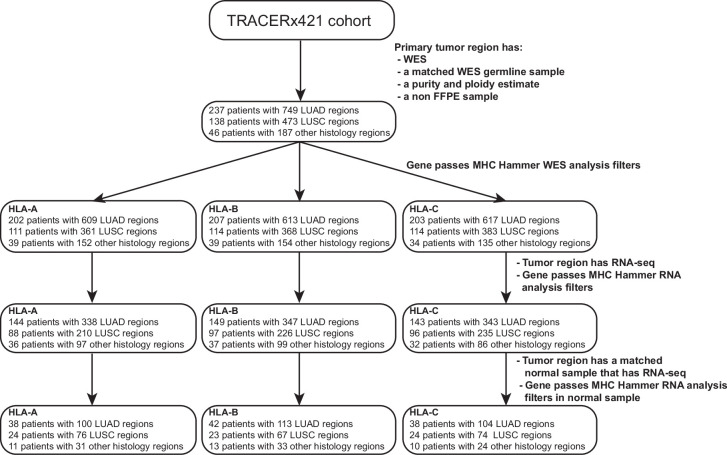
The TRACERx421 cohort. Consort diagram outlining the TRACERx421 samples used in this study. The MHC Hammer WES and RNA-seq analysis filters are outlined in the Methods. FFPE, formalin-fixed paraffin-embedded; WES, whole-exome sequencing; RNA-seq, RNA sequencing; LUAD, lung adenocarcinoma; LUSC, lung squamous cell carcinoma.

**Extended Data Fig. 2:**
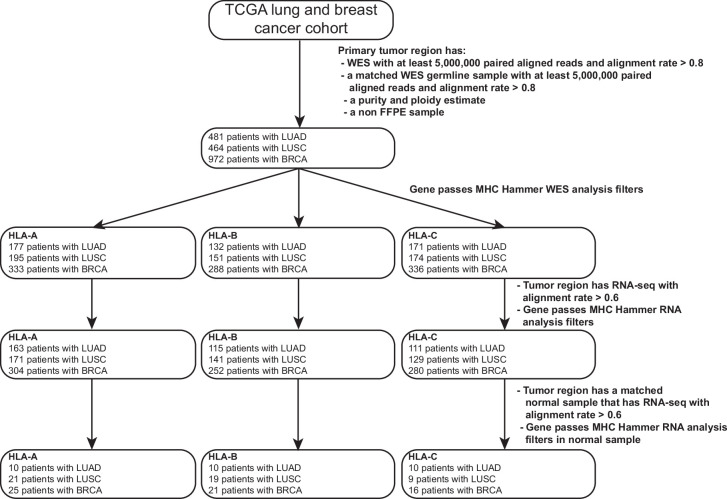
The TCGA lung and breast cancer cohort. Consort diagram outlining the TCGA lung and breast samples used in this study. The MHC Hammer WES and RNA-seq analysis filters are outlined in the Methods. FFPE: formalin-fixed paraffin-embedded; WES: whole-exome sequencing; RNA-seq: RNA sequencing; LUAD: lung adenocarcinoma; LUSC: lung squamous cell carcinoma; BRCA, breast cancer.

**Extended Data Fig. 3:**
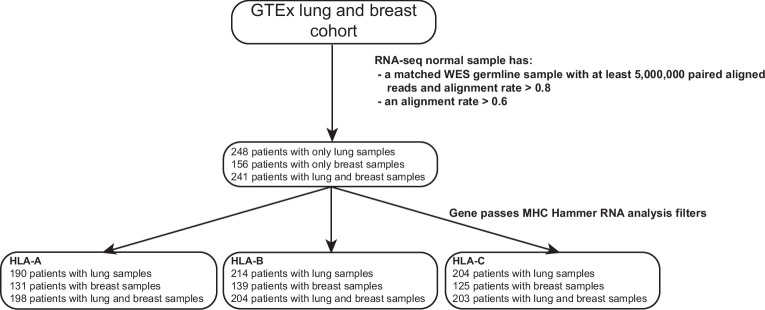
The GTEx cohort. Consort diagram outlining the GTEx lung and breast samples used in this study. The MHC Hammer RNA-seq analysis filters are outlined in the Methods. WES: whole-exome sequencing; RNA-seq: RNA sequencing.

Here we present major histocompatibility complex loss of heterozygosity, allele-specific mutation and measurement of expression and repression (MHC Hammer), a computational toolkit to accurately determine allele-specific mutations, LOH, allelic expression, allelic repression and alternative splicing of the class I HLA genes. We use MHC Hammer to investigate HLA expression in normal tissue and to evaluate genomic and transcriptomic disruption in tumor evolution in multiple cohorts, including 421 patients with NSCLC in the multiregional TRACERx421 dataset^14–16^ (Extended Data Fig. 1), 945 patients with NSCLC and 972 patients with breast cancer in the Cancer Genome Atlas (TCGA) dataset^17–19^ (Extended Data Fig. 2) and 489 normal lung and 397 normal breast samples from the Genotype-Tissue Expression (GTEx) dataset^20^ (Extended Data Fig. 3).

## Results

### A pipeline to evaluate HLA disruption

**Fig. 1:**
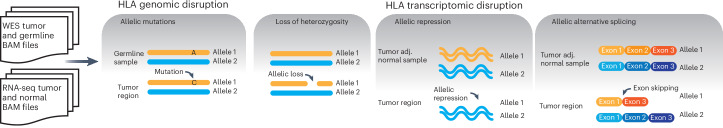
MHC Hammer: a tool to evaluate HLA DNA and RNA disruption. MHC Hammer assesses allelic mutations, LOH, allelic repression and allelic alternative splicing in the class I HLA genes from WES and RNA-seq data. tumor adj, tumor adjacent; WES, whole-exome sequencing; RNA-seq, RNA sequencing.

To evaluate the extent of genomic and transcriptomic HLA disruption, we developed MHC Hammer, advancing our LOHHLA algorithm^7^ (Fig. 1). The tool has the following four major components: (1) identifying allele-specific HLA somatic mutations, (2) calculating HLA LOH, (3) evaluating HLA allele-specific repression and (4) identifying allele-specific HLA alternative splicing. MHC Hammer is provided as a Nextflow pipeline (https://github.com/McGranahanLab/mhc-hammer) (Methods; Supplementary Note and Supplementary Fig. 1).

### HLA allele-specific expression in normal tissue

We first evaluated HLA allelic expression and alternative splicing in normal lung and breast tissue using data from the GTEx project^20^. This dataset includes 489 lung and 397 breast tissue samples from 645 healthy individuals, of which 241 have both lung and breast tissue samples available (Methods; Extended Data Fig. 3).

**Fig. 2:**
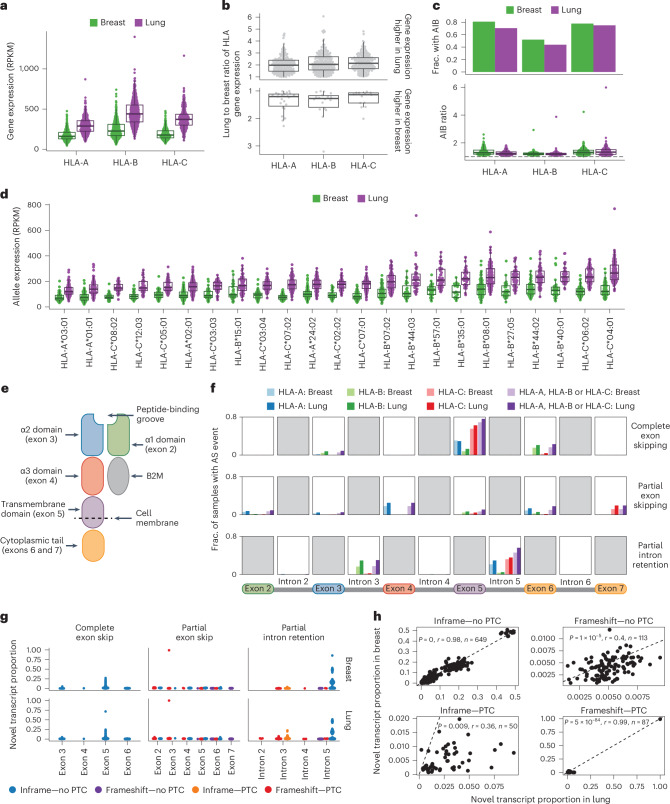
HLA expression is variable in normal tissue. **a**, Gene level expression in the GTEx lung and breast normal tissue samples for HLA-A, HLA-B and HLA-C (lung, *n* = 483 and breast, *n* = 392). **b**, The ratio of the lung to breast HLA gene expression (*n* = 238 patients with both a lung and breast sample). **c**, The fraction of tumor-adjacent normal samples with RNA AIB (top) and for the samples with AIB, the AIB ratio (bottom; lung, *n* = 440 and breast, *n* = 380). **d**, The allelic expression per allele type in lung and breast tissue. Only alleles with at least 30 lung and/or breast samples are included in this analysis (lung, *n* = 465 and breast, *n* = 377). **e**, The structure of the class I HLA molecule. **f**, Rates of alternative splicing in the GTEx normal lung and breast samples for the exons and introns shown along the bottom. **g**, The novel transcript proportion of the alternative splicing events. **h**, The relationship between the novel transcript proportion in the lung and breast tissues, for alternative splicing events found in both the lung and breast tissues of the same GTEx individual. The *P* value and correlation coefficient (*r*) in **h** are calculated using Pearson’s method. Boxplots in **a**–**d** show the median and first and third quartiles, and whiskers extend to 1.5× IQR above and below the IQR. IQR, interquartile range; AIB, allelic imbalance; AS, alternative splicing; PTC, premature termination codon; RPKM, reads per kilobase per million.

We found that in both normal lung and breast tissue, HLA-B had the highest median expression (lung, 440.3 and breast, 227.4 reads per kilobase million(RPKM)), followed by HLA-C (lung, 371.9 and breast, 177.1 RPKM), then HLA-A (lung, 289.4 and breast, 162.0 RPKM; Fig. 2a). A wide range of HLA expression was observed across the three class I genes (Fig. 2a). In individuals with both lung and breast tissue samples, HLA gene expression was higher in lung tissue in 175/198 (88%), 186/204 (91%) and 190/203 (94%) of cases for HLA-A, HLA-B and HLA-C, respectively (Fig. 2b).

Significant HLA allelic imbalance (AIB) was pervasive: 273/388 (70%), 183/418 (44%) and 305/407 (75%) of normal lung tissue samples exhibited statistically significant AIB in expression in HLA-A, HLA-B and HLA-C, respectively, while 266/329 (81%), 178/343 (52%) and 255/328 (78%) of normal breast tissue samples exhibited statistically significant AIB expression in HLA-A, HLA-B and HLA-C, respectively (Fig. 2c and Supplementary Note).

This observed expression imbalance may be due in part to the combination of HLA alleles that an individual harbors. Consistently, we observed evidence of a relationship between allelic expression and the allele type, consistent with previous reports^21,22^ (*P* < 2 × 10^−16^, one-way analysis of variance; Fig. 2d). From the alleles for which we had >30 GTEx individuals with lung and/or breast samples, we found that in lung, HLA-A*03:01, HLA-B*15:01 and HLA-C*08:02 had the lowest expression across the three genes, while HLA-A*24:02, HLA-B*40:01 and HLA-C*04:01 had the highest. In breast, HLA-A*03:01, HLA-B*15:01 and HLA-C*07:02 had the lowest expression across the three genes, whereas HLA-A*24:02, HLA-B*08:01 and HLA-C*04:01 had the highest.

### HLA alternative splicing in normal tissue samples

**Extended Data Fig. 4:**
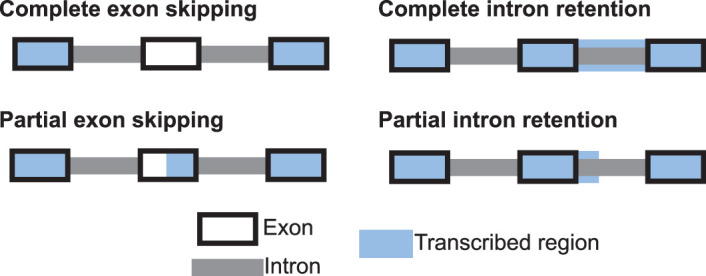
Types of alternative splicing detected by MHC Hammer. MHC Hammer will detect 4 different types of alternative splicing: complete exon skipping, complete intron retention, partial exon skipping and partial intron retention.

Given the role that HLA alternative splicing could have in HLA presentation, we first used MHC Hammer to investigate the prevalence of HLA alternative splicing in the GTEx normal tissue cohort. In our cohort, MHC Hammer identified complete exon skipping, partial exon skipping and partial intron retention in the HLA alleles, but we did not observe evidence for complete intron retention in any HLA allele (Extended Data Fig. 4 and Supplementary Note).

Alternative splicing in the HLA alleles was frequent in the GTEx normal tissue cohort; 466/483 (97%) of normal lung and 339/392 (87%) of normal breast samples harbored at least one alternative splicing event. Exon 5 skipping was the most frequent event in both lung and breast tissue, occurring in 368/483 (76%) of lung and 270/392 (69%) of breast samples, followed by partial retention of intron 5, occurring in 271/483 (56%) of lung and 180/392 (46%) of breast tissue samples (Fig. 2e,f). Skipping of exon 5 has been shown to result in a soluble HLA molecule due to the absence of the transmembrane domain^10–12^. We also observed HLA alternative splicing events in exons or introns 2, 3 or 4, which could result in an unstable HLA molecule potentially unable to present antigens to the immune system^8,9,13^ in 267/483 (55%) of normal lung tissue samples and 147/392 (38%) of normal breast tissue samples (Fig. 2e,f).

To estimate the relative abundance of the novel (alternatively spliced) transcripts, we estimated a ‘novel transcript proportion’ (Supplementary Note). Most splicing events occurred with a low novel transcript proportion, with 1,667/1,863 (90%) events in the lung and 956/1,118 (86%) events in the breast occurring with a novel transcript proportion of less than 0.1 (Fig. 2g).

**Extended Data Fig. 5:**
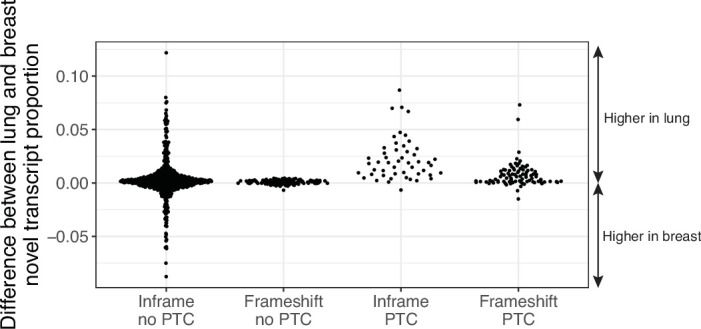
The difference between the lung and breast novel transcript proportion. For alternative splicing events that were found in both the lung and breast tissue of the same patient, the difference in the novel transcript proportion between the two tissues is shown.

We next compared the splicing events in breast and lung tissues from the same patient. In total, 43% of all alternative splicing events occurred in both breast and lung tissue, while 46% occurred only in the lung sample and just 11% occurred only in the breast sample. When restricting to events that occurred in both tissues from the same individual, we observed a high concordance between the novel transcript proportions (Fig. 2h). However, for the majority of the alternative splicing events that introduced a premature termination codon (PTC), the novel transcript proportion was higher in lung than in breast tissue (Extended Data Fig. 5). This observation could possibly be driven by differences in the rate of nonsense mediated decay (NMD) in different tissues^23,24^.

Finally, we investigated whether certain alleles were enriched for specific alternative splicing events. We focused on alternative splicing events in the 23 HLA alleles that were present in >30 GTEx individuals with breast and/or lung tissue. In lung, 10/105 alternative splicing events in these 23 alleles occurred in more than 50% of samples with the allele. In breast, 8/81 of alternative splicing events occurred in more than 50% of samples with the allele. For example, inframe complete exon 5 skipping in HLA-C*04:01:01:01 occurred in 100% of breast and lung samples (lung: 94/94, breast: 64/64) with this allele, which supports the results described in a previous study^12^. We also observed inframe partial intron 5 retention in HLA-C*03:04:01:01 in 100% of breast and lung samples with this allele (lung, 70/70 and breast, 57/57; Supplementary Table 1).

These data suggest that the HLA alleles are subject to widespread expression imbalance and alternative splicing in normal tissue and that total HLA gene expression is strongly influenced by the combination of HLA alleles that a person harbors. These data emphasize the importance of controlling for HLA allelic expression in normal tissue when assessing transcriptional alterations in tumors.

### HLA genomic disruption in lung and breast cancer

**Extended Data Fig. 6:**
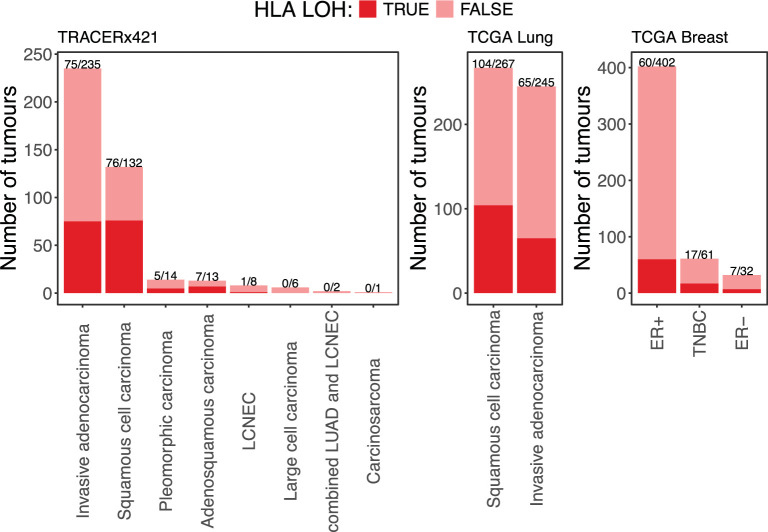
HLA LOH rates in lung and breast cancer. The rate of HLA LOH in the TRACERx421 lung cohort and the TCGA lung and breast cancer cohorts. LOH: loss of heterozygosity; LUAD: lung adenocarcinoma; LCNEC: large cell neuroendocrine carcinoma; ER+: estrogen receptor positive; ER-: estrogen receptor negative; TNBC: triple-negative breast cancer.

In the TRACERx421 cohort, LOH of the class I HLA genes was frequent, occurring in 75/235 (32%) of lung adenocarcinoma (LUAD), 76/132 (58%) of lung squamous cell carcinoma (LUSC) and 13/44 (30%) of other NSCLC histological subtype primary tumors, consistent with our previous findings^7^ (Extended Data Fig. 6). In keeping with this, the rate of HLA LOH in the TCGA lung cohort was 65/245 (27%) for LUAD tumors and 104/267 (39%) for LUSC tumors (Extended Data Fig. 6). In TCGA, triple-negative breast cancer (TNBC) had the highest rate of HLA LOH (17/61 (28%)), followed by estrogen receptor negative (ER−; 7/32 (22%)) and estrogen receptor positive (ER+; 60/402 (15%); Extended Data Fig. 6). By contrast, high-impact damaging mutations in the HLA genes were relatively rare, occurring in only 5/411 (1.2%) tumors in the TRACERx421 cohort, 2/514 (0.4%) tumors in the TCGA breast cohort and were not observed in the TCGA lung cohort.

### Transcriptional repression of class I HLA alleles in tumors

We next investigated whether there was evidence of additional disruption of HLA alleles through transcriptional repression in tumor regions. Given the heterogeneity observed in normal HLA allelic expression, we measured tumor HLA repression with reference to the patient-matched tumor-adjacent normal sample (Supplementary Note). We were able to evaluate transcriptional repression in 49 LUAD and 29 LUSC tumors in the TRACERx421 cohort and in 13 LUAD, 27 LUSC and 34 ER+ breast cancers from the TCGA cohort. We did not detect any high-impact damaging HLA mutations in any tumor with a tumor-adjacent normal sample.

**Fig. 3:**
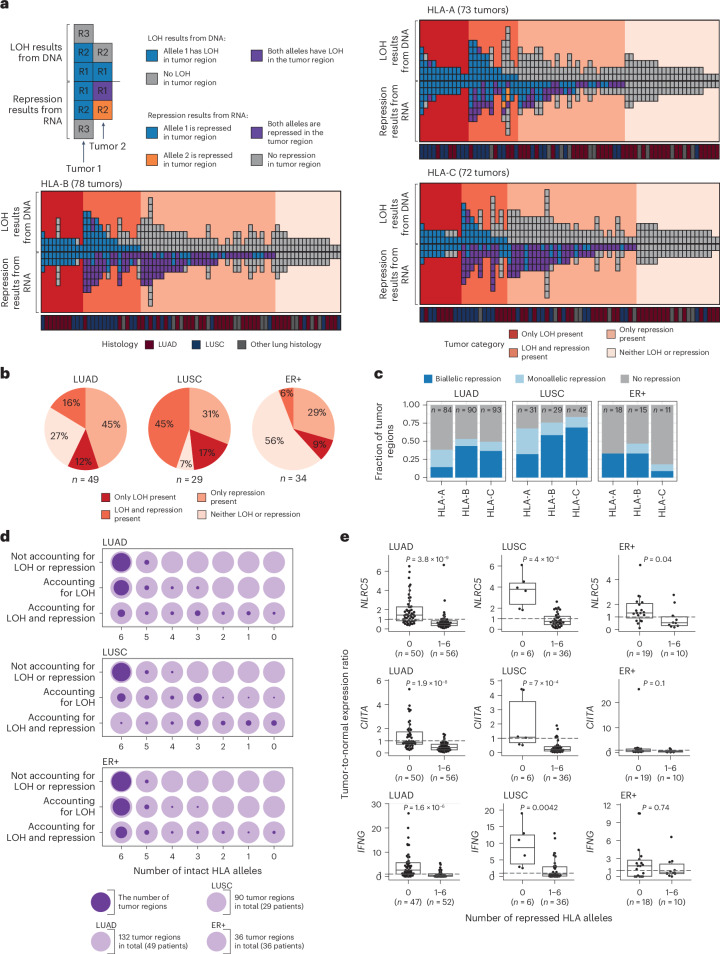
Transcriptional repression of the HLA genes in lung and ER+ breast cancer. **a**, Each column represents a tumor, and each box represents a region from that tumor. Each region appears as two boxes. The first top box (above line) is coloured by the regional HLA LOH status, and the second bottom box (mirrored below line), colored by whether the region has transcriptional repression of the same allele that is lost in the DNA (blue), the alternate allele (allele 2, orange) or both alleles (purple). Only tumors with a patient-matched tumor-adjacent normal sample are included in this figure. None of these tumors had a high-impact damaging HLA mutation. **b**, The fraction of tumors with either only HLA LOH, only repression (unexplained by genomic alterations), both HLA LOH and repression (unexplained by genomic alterations) or no HLA LOH or repression. **c**, The frequency of monoallelic and biallelic repression events in tumor regions without genomic HLA alterations. **d**, The total number of intact alleles when accounting for alleles disrupted by LOH and repression. The lighter circle indicates the number of tumor regions in total, and the superimposed darker circle indicates the number of tumor regions in the given category. **e**, The relationship between the tumor-to-normal ratio of *NLRC5*, *CIITA* and *IFNG* expression and the number of transcriptionally repressed alleles in the tumor region. The *P* value in **e** is derived from a two-sided Wilcoxon test. Boxplots in **e** show median and first and third quartiles, and whiskers extend up to 1.5× IQR above and below the IQR. LOH, loss of heterozygosity.

We identified extensive transcriptional repression of the HLA alleles that could not be explained by LOH or damaging mutations in both the lung and breast tumors (Fig. 3a,b and Supplementary Fig. 2). In the TRACERx421 cohort, 30/49 (61%) of LUAD and 22/29 (76%) of LUSC tumors harbored transcriptional repression of at least one HLA allele not caused by LOH. Taken together, just 13/49 (27%) of LUAD and 2/29 (7%) of LUSC tumors exhibited no LOH or repression in any class I HLA gene (Fig. 3b). These results were consistent in the TCGA LUAD and LUSC cohorts (Supplementary Fig. 2). In contrast, 19/34 (56%) of ER+ breast cancers exhibited no damaging mutations, LOH or repression in any class I HLA gene (Fig. 3b).

HLA genomic biallelic loss (that is, homozygous deletion) was an uncommon event, occurring in only 11/411 (3%) of TRACERx421 NSCLCs, 7/512 (1%) of TCGA NSCLCs and 17/514 (3%) of TCGA breast cancers. To investigate biallelic transcriptional repression, we restricted our analysis to HLA genes with no evidence for genomic alterations. Biallelic transcriptional repression of a given HLA gene was relatively frequent, occurring in 24/43 (56%), 11/16 (69%) and 9/32 (28%) of LUAD, LUSC and ER+ breast tumors (Fig. 3c). However, while homozygous deletion will necessarily impact both alleles equally, we found evidence of unequal biallelic repression of alleles, with 14/24 (58%), 8/11 (73%) and 4/9 (44%) of LUAD, LUSC and ER+ tumors harboring at least one HLA gene with AIB in the tumor but not the normal (or vice versa).

### The impact of HLA disruption on neoantigen presentation

**Extended Data Fig. 7:**
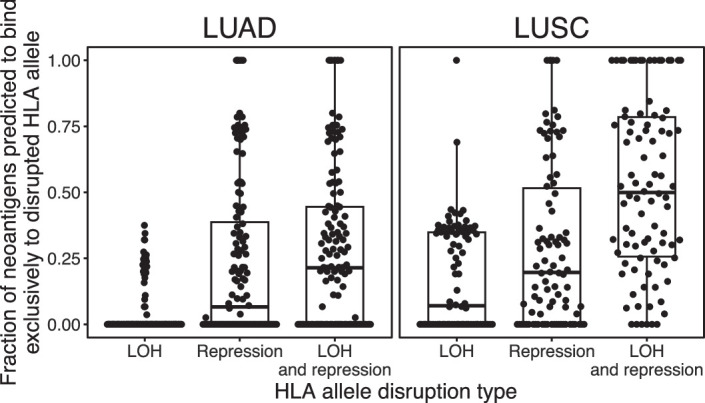
Neoantigen silencing due to HLA LOH and repression. For each tumor region, the fraction of putative neoantigens predicted to bind exclusively to HLA alleles subject to LOH and/or repression. Boxplot shows median and first and third quartiles, and whiskers extend up to 1.5× IQR above and below the IQR. LOH: loss of heterozygosity; LUAD: lung adenocarcinoma; LUSC: lung squamous cell carcinoma.

To investigate the impact of HLA LOH and transcriptional repression on the predicted number of neoantigens presented to the immune system, we quantified, for each tumor region, the number of different alleles when considering (1) neither LOH nor repression, (2) LOH or (3) LOH and repression. When accounting for LOH and repression, 39/132 (30%) of LUAD tumor regions, 3/90 (3%) of LUSC tumor regions and 18/36 (50%) of ER+ breast tumors had all six intact HLA alleles, while 9/132 (7%) of LUAD tumor regions, 18/90 (20%) of LUSC tumor regions and 3/36 (8%) of ER+ breast tumors had all six alleles disrupted at the genomic and transcriptomic levels (Fig. 3d). On average, 28.2% and 52.3% of putative neoantigens were predicted to bind exclusively to alleles subject to LOH or repression in LUAD and LUSC, respectively (Extended Data Fig. 7).

### Mechanisms of HLA repression

**Extended Data Fig. 8:**
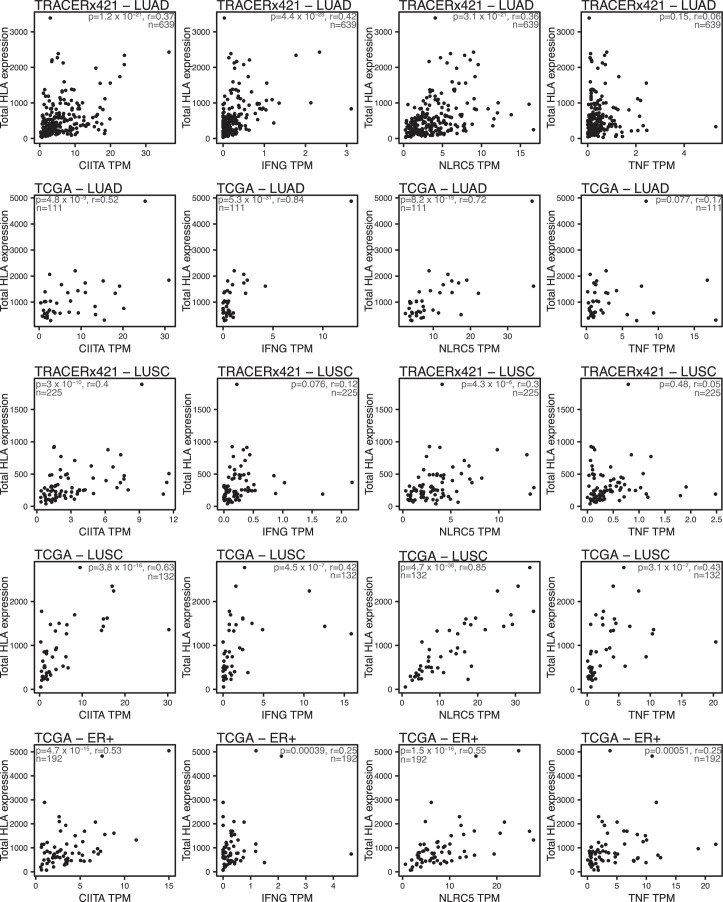
The HLA gene regulators and HLA expression. The relationship between total HLA expression, measured by MHC Hammer, and the expression of *CIITA*, *IFNG*, *NLRC5* and *TNFα* (*TNF*) in tumor samples without HLA genomic disruption. The P value and correlation coefficient (r) are calculated using Pearson’s method. TPM, transcripts per million; LUAD, lung adenocarcinoma; LUSC, lung squamous cell carcinoma; ER+, estrogen receptor positive.

The predominant modulators of HLA class I transcription are the NOD-like receptor (NLR) proteins NLRC5 and CIITA. The HLA promoter region also contains the tumor necrosis factor (TNF)-stimulated promoter site, EnhA and the IFNG-stimulated response element, ISRE^25^. In tumors without any genomic HLA disruption, we observed a significant positive correlation (Pearson’s *r* ≥ 0.3 and *P* ≤ 0.01), between total HLA expression and the expression of *NLRC5* and *CIITA* in LUAD, LUSC and ER+ tumors from both the TRACERx421 and TCGA cohorts, as well as between total HLA expression and *IFNG* expression in the TRACERx LUAD and TCGA LUAD and LUSC cohorts. We only observed a significant positive correlation between total HLA expression and *TNF* in the TCGA LUSC cohort (Extended Data Fig. 8).

We next investigated whether there was a relationship between tumor–normal changes in the expression of these genes and the likelihood of the tumor region having allelic repression unexplained by genomic disruption. In LUAD, LUSC and ER+ breast cancer, samples with allelic transcriptional repression had a significantly lower tumor-to-normal ratio of *NLRC5* than those without transcriptional repression. The same was true for *CIITA* and *IFNG* in LUAD and LUSC tumors, but not ER+ breast tumors (Fig. 3e). We only observed a significant relationship with *TNF* in LUADs (Supplementary Fig. 3).

Previous work has identified methylation as a mechanism that can influence HLA allelic expression^21,26^. To investigate the role of hypermethylation in HLA transcriptional repression, we used methylation array data from the TCGA cohorts. We observed distinct patterns of methylation across the HLA genes in the LUAD, LUSC and breast tumors, with the gene body having the highest methylation in HLA-A and HLA-B in both tumor and normal tissues. In HLA-C, the region 1,500–200 bp upstream of the transcriptional start site (termed TSS1500) had the highest level of methylation, followed by the gene body, in both the tumor and normal tissues (Supplementary Fig. 4). We observed a significant negative correlation (Pearson’s *r* ≤ −0.3 and *P* ≤ 0.01) between HLA-B gene expression and the degree of methylation in both the TSS1500 and the gene body region in the TCGA LUAD, LUSC and ER+ tumor samples (Supplementary Fig. 5–7).

Taken together, these data suggest that in the LUAD, LUSC and breast tumors, changes in the expression of *NLRC5*, *CIITA*, *IFNG* as well as hypermethylation may play a role in the repression of HLA.

### HLA alternative splicing in breast and lung cancer

**Fig. 4:**
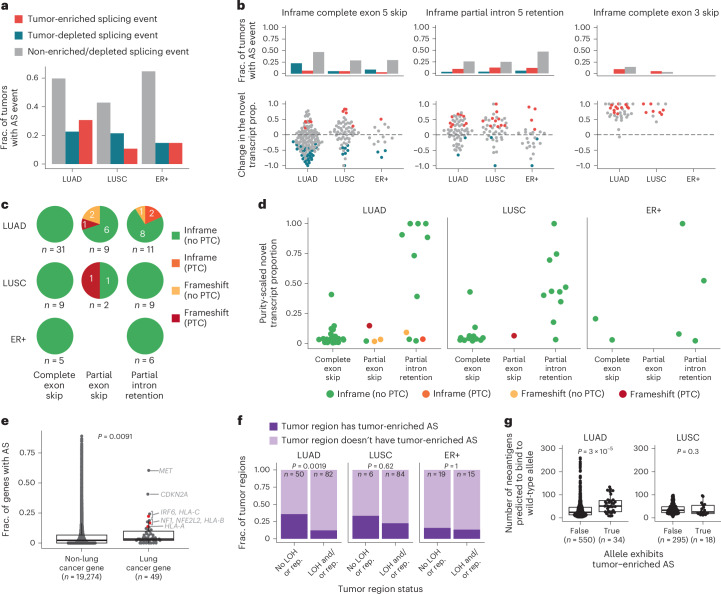
HLA alternative splicing in lung and breast tumors. **a**, The fraction of tumors that exhibit tumor-enriched or tumor-depleted HLA alternative splicing events in the TRACERx (LUAD and LUSC) and TCGA (ER+) cohorts. **b**, The three most frequent HLA alternative splicing events are shown. The fraction of tumors that exhibit the event is shown at the top and the tumor-to-normal change in the novel transcript proportion is shown at the bottom. The legend is shown in **a**. **c**, The predicted consequences of the tumor-enriched and tumor-depleted alternative splicing events. **d**, The purity-scaled novel transcript proportion for the tumor-enriched alternative splicing events. **e**, The fraction of TRACERx tumors that exhibit alternative splicing events across all protein-coding genes, split by whether the gene is classified as a lung cancer gene or not. The three HLA genes are shown in red. **f**, The fraction of tumor regions that do/do not have LOH or repression and do/do not have tumor-enriched alternative splicing events. **g**, The neoantigen count for each allele is split by whether the allele exhibits tumor-enriched alternative splicing or not. Only alleles with no genomic disruption were included. *P* values in **e** and **g** are derived from a two-sided Wilcoxon test. *P* value in **f** is derived from Fisher’s exact test. Boxplots in **e** and **g** show median and first and third quartiles, and whiskers extend up to 1.5× IQR above and below the IQR. AS, alternative splicing; LOH, loss of heterozygosity; TSG, tumor suppressor gene; PTC, premature termination codon; rep, repression.

Given the pervasive nature of alternative splicing in normal tissue, we evaluated whether splicing events occurred at significantly higher frequency in the tumor (‘tumor-enriched’) or, conversely, at significantly lower frequency in the tumor (‘tumor depleted’; Supplementary Note). Both tumor-enriched and tumor-depleted HLA alternative splicing events were common, with 30.6%, 10.7% and 14.7% of LUAD, LUSC and ER+ breast tumors harboring at least one tumor-enriched alternative splicing event and 22.6%, 21.4% and 14.7% of LUAD, LUSC and ER+ tumors harboring at least one tumor-depleted alternative splicing event (Fig. 4a).

The most frequent tumor-enriched alternative splicing events included inframe partial intron 5 retention and inframe complete exon 3 skipping (Fig. 4b). Changes to the sequence that encodes exon 3 could result in altered peptide binding or an unstable HLA molecule^8,13^. In contrast, inframe complete exon 5 skipping was observed more frequently as a tumor-depleted event in LUAD and ER+ tumors (Fig. 4b). Skipping of exon 5 has been demonstrated in previous studies to result in a soluble HLA molecule^10–12^. For each alternative splicing event, the tumor-to-normal change in the novel transcript proportion is shown (Supplementary Note and Fig. 4b).

The introduction or deletion of nucleotide sequences due to alternative splicing could result in a frameshift and/or the introduction of a PTC in the resulting transcript. In LUAD, LUSC and ER+ tumors, all complete exon skipping events were inframe (Fig. 4c). In contrast, we observed partial exon skipping events and partial intron retention events that were inframe, or that resulted in a frameshift, with and without the introduction of a PTC (Fig. 4c).

Given that tumor samples reflect an admixture of cancer cells and non-cancer cells, to estimate the fraction of alternatively spliced transcripts in the cancer cells, we scaled the novel transcript proportion of the tumor-enriched events by the estimated purity of the tumor region (Supplementary Note). Although there were outliers, the purity-scaled novel transcript proportion was less than 0.25 in most cases (mean = 0.24 and range = 0.006–1; Fig. 4d). These data suggest either one or both of the following are occurring: within each cancer cell, both the canonical and novel transcripts are being transcribed, or only a subset of cancer cells harbor the novel transcript.

To further evaluate the rate of tumor-enriched alternative splicing observed in HLA alleles and whether this is higher or lower than expected, we considered the rate of somatic alternative splicing across all protein-coding genes (Methods). We found that lung cancer genes had a higher rate of alternative splicing than other protein-coding genes (*P* = 9.1 × 10^−3^; Fig. 4e). In addition, from the set of 49 lung cancer genes, we found that HLA-C had the fourth, HLA-B had the seventh and HLA-A had the eighth highest frequency of alternative splicing (Fig. 4e).

Consistent with the selection of alternative splicing events, we observed that LUAD tumor regions without HLA LOH or repression were enriched for tumor-enriched alternative splicing events (LUAD, *P* = 1.9 × 10^−3^) compared to regions that harbored either HLA LOH or repression (Fig. 4f). We did not see this enrichment with LUSC or ER+ tumors. This suggests that in LUAD, tumor-enriched alternative splicing may offer an alternative means to disrupt HLA presentation during tumor evolution.

To further investigate the importance of HLA alternative splicing in tumor evolution, we compared the total number of neoantigens predicted to bind to alleles with or without tumor-enriched HLA alternative splicing. We first quantified the number of neoantigens predicted to bind to the intact HLA alleles for each tumor region. We then compared the neoantigen count in alleles that exhibited tumor-enriched alternative splicing versus those that did not, excluding alleles with HLA LOH. HLA alleles exhibiting tumor-enriched HLA alternative splicing in LUAD tumors were associated with a higher neoantigen count compared to alleles without evidence of tumor-enriched splicing (*P* = 3 × 10^−5^; Fig. 4g), suggesting that in LUAD tumors, tumor-enriched alternative splicing of HLA alleles may be selected to reduce antigen presentation. We did not observe this enrichment in LUSC tumors.

### HLA disruption and tumor evolution

**Fig. 5:**
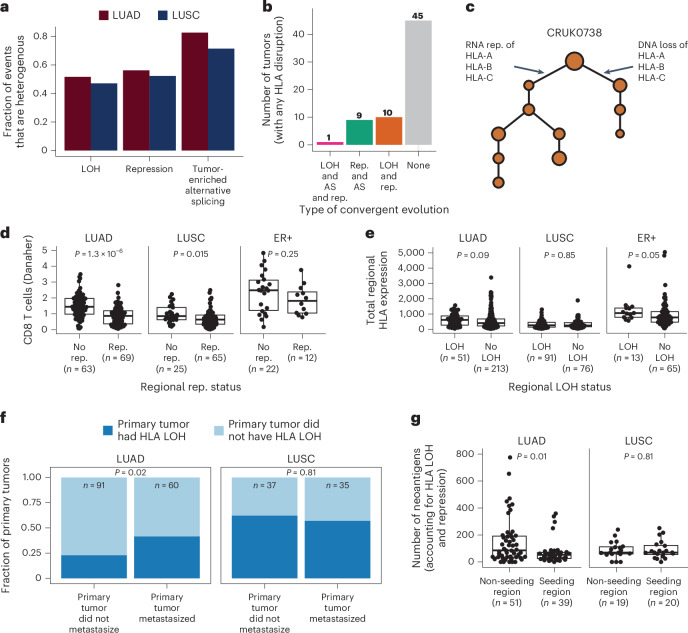
The role of HLA disruption in tumor evolution. **a**, The heterogeneity of HLA LOH, repression and tumor-enriched alternative splicing events. **b**,**c**, Overview (**b**) and example (**c**) of convergent evolution, where the same HLA allele is disrupted via different mechanisms in different regions of the same tumor. **d**, The relationship between the presence of repression and the amount of CD8 T cell infiltration. **e**, Tumor regions with and without HLA LOH have similar levels of total HLA expression. **f**, LUAD tumors that have HLA LOH are more likely to metastasize. **g**, When accounting for LOH and repression, LUAD regions that seeded a metastasis have a lower neoantigen count than those that did not. *P* values in **d**, **e** and **g** are derived from a two-sided Wilcoxon test. *P* value in **f** is derived from Fisher’s exact test. Boxplots in **d**, **e** and **g** show median and first and third quartiles, and whiskers extend up to 1.5× IQR above and below the IQR. LOH, loss of heterozygosity; rep, repression; AS, alternative splicing.

To understand when HLA LOH, transcriptional repression and somatic alternative splicing occur during NSCLC evolution, we considered the heterogeneity of these events using the multiregion TRACERx cohort. We defined an HLA disruption event as ubiquitous if it occurred in all of the primary tumor regions, and heterogeneous otherwise. In both LUAD and LUSC tumors, tumor-enriched alternative splicing events were the most heterogeneous (LUAD = 82.6% and LUSC = 71.4%), followed by repression (LUAD = 56.2% and LUSC = 52.2%) and then LOH events (LUAD = 51.6% and LUSC = 47.1%; Fig. 5a).

In 20/65 (30.8%) of TRACERx LUAD and LUSC tumors with HLA disruption, we observed convergence upon disruption of the same allele through alternative mechanisms, with genomic loss, transcriptional repression and/or alternative splicing of the same allele occurring in different regions of the same tumor. We observed ten tumors with convergence upon genomic loss and transcriptional repression of the same allele in separate regions, nine tumors with transcriptional repression and alternative splicing of the same allele in separate regions and one tumor with genomic loss, transcriptional repression and alternative splicing of the same allele in different regions (Fig. 5b,c). Conceivably, this could either reflect positive selection within individual tumors or be a consequence of the high rate of HLA disruption through diverse mechanisms.

**Extended Data Fig. 9:**
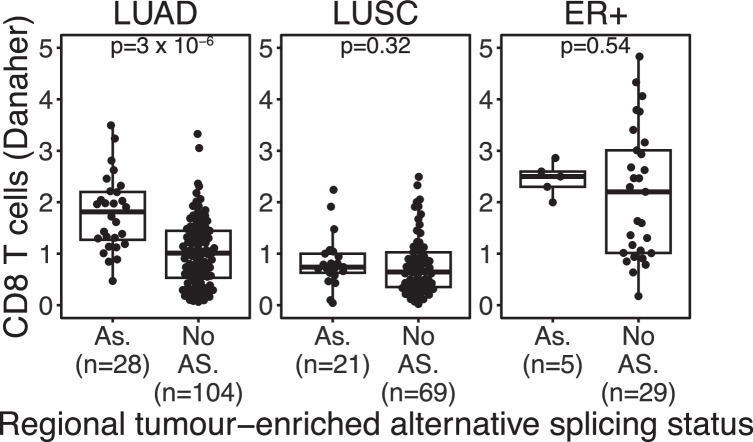
CD8 T cells and HLA alternative splicing. The relationship between the presence of tumor-enriched alternative splicing (AS) and the amount of CD8 T cell infiltration. P values were derived from a two-sided Wilcoxon test. Boxplot shows median and first and third quartiles, and whiskers extend up to 1.5× IQR above and below the IQR. LUAD: lung adenocarcinoma; LUSC: lung squamous cell carcinoma; ER+: estrogen receptor positive; AS: alternative splicing.

The tumor microenvironment can shape tumor evolution^1^. We therefore investigated the relationship between the immune infiltrate and the presence of HLA disruption, using the Danaher in silico immune deconvolution method to estimate the amount of CD8 T cell infiltrate^27^. We observed a significant relationship between total HLA expression and CD8 T cell infiltrate (LUAD, *P* = 1.6 × 10^−27^ and *r* = 0.48; LUSC, *P* = 3.7 × 10^−15^ and *r* = 0.44; ER+, *P* = 1.3 × 10^−24^ and *r* = 0.42; Supplementary Fig. 8). We observed that LUAD and LUSC tumor regions with allelic HLA transcriptional repression had lower levels of infiltrating CD8 T cells compared to those without (LUAD, *P* = 1.3 × 10^−6^; LUSC, *P* = 0.015 and ER+, *P* = 0.25; Fig. 5d). Conversely, HLA alternative splicing was associated with elevated CD8 T cell levels in LUAD tumors (LUAD, *P* = 3 × 10^−6^; LUSC, *P* = 0.32 and ER+, *P* = 0.54; Extended Data Fig. 9). No clear relationship between HLA LOH and total HLA expression in either LUAD, LUSC or ER+ tumors was observed, indicating dosage compensation may occur following allelic HLA copy number loss (Fig. 5e).

**Extended Data Fig. 10:**
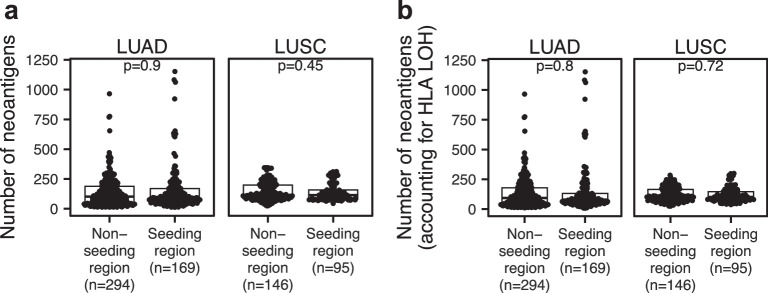
Tumor neoantigen burden and metastasis seeding. **a**, The neoantigen count of primary tumor regions, split by whether they did or did not seed a metastasis. **b**, The neoantigen count of primary tumor regions, restricted to reflect peptides binding only to HLA alleles without HLA LOH, split by whether the primary tumor regions did or did not seed a metastasis. P values were derived from a two-sided Wilcoxon test. Boxplot shows median and first and third quartiles, and whiskers extend up to 1.5× IQR above and below the IQR. LOH: loss of heterozygosity; LUAD: lung adenocarcinoma; LUSC: lung squamous cell carcinoma.

Finally, we endeavored to understand whether disruption of the HLA alleles through LOH, repression or alternative splicing might have a role in the evolution of lung cancer metastasis. We found that LUAD tumors harboring HLA LOH were more likely to metastasize than those without HLA LOH (LUAD, *P* = 0.02 and LUSC, *P* = 0.81; Fig. 5f). To investigate this further, we considered the neoantigen burden of primary tumor regions that seeded metastasis compared to those that did not, with the metastasis-seeding regions being defined in our previously published work^15^. We found that the standard neoantigen burden did not distinguish seeding from non-seeding regions (Extended Data Fig. 10a). However, when we restricted our neoantigen count to only include neoantigens predicted to bind to intact HLA alleles, not subject to loss or repression, we observed that LUAD tumor regions that seeded metastasis had a lower effective neoantigen burden than those that did not (LUAD, *P* = 0.01 and LUSC, *P* = 0.81; Fig. 5g), which was not the case when we only considered HLA loss (Extended Data Fig. 10b). Taken together, these data suggest that disruption of the HLA alleles could have an important role in tumor metastasis.

## Discussion

Neoantigen presentation via HLA molecules is crucial to achieving an antitumor immune response. Previous studies have illustrated that different mechanisms of HLA disruption are common across cancers^4–7^. Here we developed MHC Hammer, a tool to investigate the prevalence of four mechanisms of genomic and transcriptomic disruption of the HLA alleles in lung and breast cancer—mutations, LOH, repression and alternative splicing.

While damaging HLA mutations were rare in our cohorts, LOH, repression and tumor-enriched alternative splicing of the HLA alleles were pervasive. From the patients with tumor-adjacent normal samples, just 27% of LUAD, 7% of LUSC and 56% of ER+ tumors had no HLA disruption, while 30.6%, 10.7% and 14.7% of LUAD, LUSC and ER+ tumors exhibited tumor-enriched alternative splicing events. The lower rate of HLA LOH and repression observed in ER+ breast tumors may reflect the lower tumor mutational burden (TMB) in ER+ breast tumors compared to NSCLC^28^.

We observed differences in the patterns of HLA disruption in the NSCLC tumors—LUSC tumors were characterized by almost universal HLA disruption, while LUAD tumors exhibited less frequent HLA disruption. In LUADs, we observed an enrichment for alternative splicing in alleles without LOH or repression, a higher likelihood of tumor-enriched alternative splicing in alleles with a higher neoantigen burden and finally an association between HLA LOH and metastasis. This may reflect different selective pressures in these cancer types and the propensity for HLA disruption through diverse mechanisms.

One limitation of our method is that it requires a patient-matched tumor-adjacent normal tissue sample to determine HLA repression and tumor-enriched alternative splicing. This is due to the high variability observed in HLA allelic expression and the high prevalence of HLA alternative splicing in the normal tissue samples.

Alternative splicing of the class I HLA alleles has been observed in non-cancer tissue cohorts and in cancer cell lines^8–13^. However, HLA alternative splicing in large cohorts of normal and tumor tissue has not been described before, due in part to the lack of a high-throughput bioinformatics tool capable of measuring HLA alternative splicing.

HLA alternative splicing affecting exons or introns 2–4 could result in an unstable HLA molecule. For example, partial exon 3 skipping in an HLA-A allele in non-cancer tissue has been shown to result in the absence of cell-surface expression^13^. An HLA-A allele with complete exon 3 skipping continued to be expressed on the cell surface but as an immature glycoprotein unable to present peptides^8^. This immature molecule could potentially act as a decoy allele by inhibiting NK cells via its receptor ligands without presenting neoantigens to CD8 T cells. Alternative splicing resulting in exon 5 skipping has been shown to result in a soluble HLA allele^10–12^. Persistent presentation of neoantigens via soluble HLA molecules to the T cell receptor, without costimulatory or accessory signals, could lead to immune tolerance or T cell exhaustion. It has been shown that soluble class I HLA molecules can induce apoptosis in CD8 T cells and NK cells^29^.

The majority of the detected tumor-enriched alternative splicing events were present with a purity-adjusted novel transcript proportion <0.25. This could reflect NMD; PTC-induced NMD has been shown to reduce mRNA levels by up to 90% in a study of an HLA-A allele^23^. Alternatively, HLA alternative splicing may be a transient event, or the alternative splicing observed in the lung and breast tumors in this study may simply reflect transcriptional noise. Therefore, further work is required to establish the role of alternative splicing in lung and breast cancer, as well as investigate its prevalence in other cancer types.

It is possible that the underlying mechanisms of HLA repression and alternative splicing events are epigenetic. Supporting this, we found a strong link between methylation and expression of the HLA genes. This could have important clinical implications, as previous studies have illustrated the reversible nature of HLA epigenetic modifications^26^ and the importance of this reversibility in immunotherapy response^30^.

Further work is warranted to explore the extent to which HLA alternative splicing and repression represent a pan-cancer immune evasion mechanism. As more pre-therapy and post-therapy data emerge, it will be possible to investigate the extent to which HLA alternative splicing and repression develop during treatment and at immune-therapy resistance and the extent to which these processes might inform therapeutic strategies.

Our results may also have implications for vaccine- and T cell-based therapeutic approaches, which seek to exploit neoantigens. Our results suggest that it may be important to consider not just whether putative neopeptides bind the repertoire of HLA alleles but also the copy number, expression and splicing characteristics of each allele. Indeed, MHC Hammer may be used to help determine which set of predicted neoantigens are most likely to elicit an effective T cell response.

In conclusion, MHC Hammer enables accurate estimation of allele-specific HLA disruption, revealing that it is a common feature of NSCLC and ER+ breast cancer that facilitates immune escape and cancer evolution.

## Methods

### The TRACERx421 data

The TRACERx421 samples used in the study have been described in previously published manuscripts^14,16^. The design of the TRACERx study has been approved by an independent research ethics committee (13/LO/1546) and the ClinicalTrials.gov number is NCT01888601. Informed consent for entry into the TRACERx study was mandatory and obtained from every patient. The purity and ploidy estimates, histological subtypes, lung cancer genes and phylogenetic trees used in this study were taken from a previous TRACERx study^16^. Transcripts per million (TPMs) estimates were taken from a previously published TRACERx study^14^. The classification of primary tumors that did and did not metastasize, as well as the classification of seeding regions, was taken from a previously published TRACERx study^15^. Only primary tumor and non-lymph node regions with purity and ploidy estimates were used in this study. The consort diagram of the TRACERx samples used in the study is shown in Extended Data Fig. 1.

### The TCGA cohort

MHC Hammer was run on lung and breast samples from the TCGA dataset^17–19^. We implemented the following thresholds for a sample to be included in our study:

- Whole-exome sequencing (WES) samples with less than 5,000,000 paired aligned reads or an alignment rate of less than 0.8 were excluded.
- RNA sequencing (RNA-seq) samples with an alignment rate of less than 0.6 were excluded.
- Formalin-fixed paraffin-embedded (FFPE) samples and metastatic samples were excluded.
- Tumor samples without a purity and ploidy solution were excluded.
- Samples without a matched WES germline sample that passed our filters were excluded.

In the cases where a TCGA sample was sequenced multiple times, we selected a single sequencing run to use in our study. For the WES tumor samples, we prioritized choosing a non-whole-genome amplification (non-WGA) sample over a WGA sample^31^, and then prioritized the samples by the number of paired and aligned reads. For the WES germline samples, we also prioritized non-WGA over WGA, then blood samples over solid tissue samples and finally prioritized the samples by the number of paired and aligned reads. For the RNA-seq samples, we chose the sample with the highest number of paired and aligned reads.

The purity and ploidy solutions for the TCGA cohort were estimated using ASCAT^32^ and taken from https://github.com/VanLoo-lab/ascat/tree/master/ReleasedData/TCGA_SNP6_hg38. The methylation array data and TPM data used in this study were downloaded from Genomic Data Commons (GDC). The lung histological subtypes (LUAD and LUSC) were taken from GDC, and the breast subtypes (ER+, ER- and TNBC) were taken from cBioPortal.

The consort diagram for the TCGA cohort is shown in Extended Data Fig. 2.

### The GTEx cohort

We ran MHC Hammer on the normal lung and breast RNA-seq samples from the GTEx dataset^20^. We implemented the following thresholds for a sample to be included in our study:

- WES germline samples with less than 5,000,000 paired aligned reads or an alignment rate of less than 0.8 were excluded.
- RNA-seq samples with an alignment rate of less than 0.6 were excluded.
- Samples without a matched WES germline sample that passed our filters were excluded.

The consort diagram for the GTEx cohort is shown in Extended Data Fig. 3.

### Validation of allele-specific HLA alternative splicing

To validate our HLA alternative splicing pipeline, we used allele-specific PCR amplification. We performed this for four tumor regions and one normal sample from two patients (CRUK0061_SU_N01, CRUK0061_SU_T1-R1, CRUK0061_SU_T1-R2, CRUK0733_SU_T1-R2 and CRUK0733_SU_T1-R6). RNA-seq data were available for four of these samples (CRUK0061_SU_N01, CRUK0061_SU_T1-R1, CRUK0733_SU_T1-R2 and CRUK0733_SU_T1-R6), and MHC Hammer identified exon 5 skipping in an HLA-C allele in all four samples.

To amplify each allele, we used allele-specific primers that have been described previously^12^, and the fragment sizes were confirmed via agarose gel electrophoresis (Supplementary Fig. 1a). These PCR products were then cloned using a TA cloning kit (Invitrogen), where the wild-type and novel alternatively spliced transcripts were subsequently validated through Sanger sequencing (Supplementary Fig. 1b).

### Neoantigen calls

Patient-specific HLA haplotype predictions were obtained using HLA-HD^33^ (version 1.2.1). NetMCHpan4.1 (ref. ^34^) was run on 9–11 neopeptides derived from nonsynonymous mutations across the TRACERx421 cohort, taking into account patient-specific HLA types. A cutoff of 0.5 in the eluted ligand rank was applied to define whether a peptide is bound to a specific HLA type. An observed nonsynonymous mutation is deemed a neoantigen binding to a specific HLA if at least one of its neopeptides is considered a binder.

### Danaher estimates of CD8^+^ T cell infiltration

The amount of CD8 T cell infiltration was estimated using the Danaher method^27^. To do this, TPM values of the *CD8A* and *CD8B* genes were first converted to log_2_, and the mean log_2_ value across the two genes was taken for each sample.

### Calling alternative splicing in all protein-coding genes

To call alternative splicing in all protein-coding genes, we used the STAR aligner with a two-pass alignment^35,36^ and the GRCh38 reference sequence to generate a set of splice junctions in the TRACERx samples. Novel splice junctions were defined as those not present in the GRCh38 RefSeq GTF file (https://hgdownload.soe.ucsc.edu/goldenPath/hg38/bigZips/genes/). To be considered as present in a tumor region, a novel splice junction required at least 20 uniquely mapping reads in any region from the tumor and at least two uniquely mapping reads in the given region. To be considered somatic, the novel splice junction could not be present in the patient-matched normal sample.

### The MHC Hammer pipeline

See Supplementary Note for a detailed overview of the MHC Hammer pipeline. The following MHC Hammer parameters were used to generate the data in this study:

- The HLA reference files were created using the ImMunoGeneTics (IMGT) database version 3.38 (ref. ^37^).
- The library size was estimated as the number of paired and aligned reads in the input BAM files (include_unmapped_reads_in_library_size = FALSE).
- The HLA FASTQ files were created by filtering the input BAM files to include all unmapped reads, reads that mapped to chromosome 6 or any alternate contig or reads that contained a 30-mer sequence from the IMGT database (unmapped_reads = TRUE, contig_reads = TRUE, fish_reads = TRUE).
- When filtering the HLA allele BAM files, reads with more than one mismatch to the patient-specific reference were removed (max_mismatch = 1).
- In the estimation of allelic copy number and DNA AIB, filtered SNPs required a read depth of at least 30 in TRACERx (min_depth = 30) or 5 in TCGA (min_depth = 5).
- After the first STAR alignment, splice junctions required at least two supporting reads to be included in the cohort of splice junctions in the second STAR alignment (uniq_num_across_junc = 2).

#### Filters implemented for HLA WES analysis

To be included in the HLA DNA analysis, including HLA copy number, AIB and LOH calls, a gene must pass the following filters:

- Have at least ten SNPs that pass the minimum read depth of 30 in the TRACERx samples or 5 in the TCGA samples.
- Both alleles of the gene must have an expected depth of at least 10. The expected depth estimates the depth of the reads that are coming solely from the cancer cells (see Supplementary Note for more details).
- The 95% confidence interval in the allelic copy number, calculated using the R function t.test, must be less than 2.5.

#### Filters implemented for somatic HLA mutations

HLA allelic mutations were classified as high-impact and damaging if the Ensembl Variant Effect Predictor (VEP)^38^ consequence included at least one of ‘stop_gained’, ‘frameshift_variant’, ‘start_lost’ or ‘stop_lost’. To be considered in our analysis, mutations had to be classified as ‘PASS’ using the Genome Analysis ToolKit (GATK)^39^ FilterMutectCalls function, have ten reads supporting the alternate allele, and fall in a sample and gene that passed the MHC Hammer WES filters.

#### Filters implemented for HLA RNA analysis

To be included in the HLA RNA analysis, including RNA AIB, allelic expression, allelic repression and alternative splicing, a gene must pass the DNA analysis filters and

- Have at least ten SNPs in the exon sequence.
- Have no more than 50% of reads mapping to both alleles of the same gene.
- Have no more than 5% of reads mapping to multiple HLA genes.

#### Filters implemented for HLA alternative splicing

We excluded from our analysis any novel splice junction detected in the first or last exons of an HLA gene. To be included in our analysis, novel splice junctions had to be classified as one of the following: complete exon skipping, partial exon skipping, partial intron retention or complete intron retention. In addition, we applied the following depth filters:

- In the GTEx cohort—to be included in our analysis, a novel splice junction required at least two uniquely mapping reads in that sample. In addition, the novel splice junction needed to be identified in another sample from the same patient with at least 20 uniquely mapping reads.
- In the TRACERx and TCGA cohorts—to be included in our analysis, a novel splice junction needed to be classified as either ‘tumor-enriched’ or ‘tumor-depleted’ and also be identified in at least one tumor region or matched normal from the patient with at least 20 uniquely mapping reads.

### Statistical information

All statistical tests were performed in R (v.4.3.3). No statistical methods were used to predetermine the sample size. Tests involving comparisons of distributions were done using a two-tailed Wilcoxon test (wilcox.test). Tests involving the comparison of groups were done using a two-tailed Fisher’s exact test (fisher.test). The correlation was tested using Pearson’s correlation coefficient (cor.test).

### Reporting summary

Further information on research design is available in the Nature Portfolio Reporting Summary linked to this article.

## Online content

Any methods, additional references, Nature Portfolio reporting summaries, source data, extended data, supplementary information, acknowledgements, peer review information; details of author contributions and competing interests; and statements of data and code availability are available at 10.1038/s41588-024-01883-8.

## Supplementary information

Supplementary Information Supplementary Note and Supplementary Figs. 1–8.

Reporting Summary

Supplementary Table 1 The frequency of alternative splicing events in common HLA alleles in the GTEx dataset.

## Data Availability

The WES and RNA-seq data used during this study have been deposited at the European Genome–Phenome Archive, which is hosted by the European Bioinformatics Institute and the Centre for Genomic Regulation under the accession code EGAS00001006494. Access is controlled by the TRACERx data access committee. Details on how to apply for access are available on the linked page. The TRACERx data are available under controlled access so that patient privacy and data confidentiality are maintained while promoting and encouraging impactful scientific discovery. The data access committee aims to reply to requests within 1 week.

## Code availability

Code to run the MHC Hammer pipeline can be found at https://github.com/McGranahanLab/mhc-hammer.

Code to recreate the manuscript figure can be found at https://zenodo.org/records/13388455 (ref. ^40^).
