# Supplementary material for: MHC Hammer reveals genetic and non-genetic HLA disruption in cancer evolution
Source: Nat Genet. 2024 Oct 2;56(10):2121–31. doi: 10.1038/s41588-024-01883-8 (PMC11525181; doi:10.1038/s41588-024-01883-8)
Supplement: Supplementary file 1 — Supplementary Note and Supplementary Figs. 1–8. [file 41588_2024_1883_MOESM1_ESM.pdf]

nature genetics

Supplementary information https://doi.org/10.1038/s41588-024-01883-8

MHC Hammer reveals genetic and non-genetic HLA disruption in cancer evolution

In the format provided by the authors and unedited

Supplementary Note: The MHC Hammer pipeline

Input files **2**
Steps in the MHC Hammer pipeline **2**
Preprocessing steps 2
Estimating the library size 2
Creating the HLA FASTQ files 2
Predicting the germline HLA allele types 3
Constructing the patient-specific HLA references 3
Alignment steps 3
Aligning the WES and RNA-seq data using NovoAlign 3
Aligning the RNA-seq data using STAR 4
Filtering the HLA allele BAM files 4
Calculating HLA SNP positions 5
DNA analysis 5
Calculating the DNA HLA allelic copy number 5
Calculating the DNA HLA allelic imbalance 5
Evaluating loss of heterozygosity 6
Calling HLA allelic somatic mutations 6
Calculating the DNA expected depth 6
RNA analysis 6
Quantifying HLA allelic expression 6
Calculating RNA allelic imbalance 7
Calculating HLA allelic repression 7
Calling HLA alternative splicing 8
References **12**

Input files

MHC Hammer requires every individual to have a whole exome sequencing (WES) germline BAM file, which is used to predict the HLA allele types. In addition, MHC Hammer requires the following:

To estimate DNA HLA allelic imbalance and somatic mutations:

- A tumour WES BAM file.

To estimate DNA HLA copy number and loss of heterozygosity (LOH):

- A tumour WES BAM file with purity and ploidy estimates.

To estimate RNA HLA allelic expression, allelic imbalance and alternative splicing:

- A tumour or normal RNA sequencing (RNA-seq) BAM file.

To estimate RNA HLA allelic repression, tumour-enriched and tumour-depleted alternative splicing:

- A tumour and normal RNA-seq BAM file. The normal sample should be from the same patient and tissue as the tumour.

Steps in the MHC Hammer pipeline

Preprocessing steps

Estimating the library size

The number of paired and aligned reads in the input BAM file is used as an estimate of the library size of the sample. Alternatively, the library size can be estimated as the number of aligned and unaligned paired reads in the input BAM file by using the MHC Hammer parameter `include_unmapped_reads_in_library_size = TRUE`.

Creating the HLA FASTQ files

To reduce the computational resources required by MHC Hammer, the input WES and RNA-seq BAM files are first filtered to keep only reads that may have originated from the HLA region. These reads are stored in a FASTQ file called the HLA FASTQ file. The following MHC Hammer parameters can be used to control which reads from the input BAM file are included in the HLA FASTQ file:

- `unmapped_reads`: can be true or false. If true, unmapped reads in the input BAM file are included in the HLA FASTQ file.
- `contig_reads`: can be true or false. If true, reads that map to either any alternative contig in the input BAM files (if the input parameter `contigs_file` is empty), or contigs specified by the user (if `contigs_file` is a file path), are included in the HLA FASTQ file.

- `contigs_file`: can be empty or the path to a file. This file should contain a list of contig names.
- `fish_reads`: can be true or false. If true, reads in the input BAM files that contain a 30-mer sequence that is also present in a sequence in the ImMunoGeneTics[1] (IMGT) database are included in the HLA FASTQ file.
- `fish_reads_only`: can be true or false. If true, only reads in the input BAM files that contain a 30-mer sequence that is also present in a sequence in the IMGT database are included in the HLA FASTQ file.
- `mhc_coords`: path to the file containing a set of genomic coordinates (chr:star-stop). Any read in the input BAM file that lies within these coordinates is included in the HLA FASTQ file.

Predicting the germline HLA allele types

MHC Hammer uses the bioinformatic tool HLA-HD[2] (v1.7.0) to predict the HLA allele types from the germline WES HLA FASTQ file. HLA-HD was chosen as it has been demonstrated as an accurate HLA typing algorithm designed for next-generation sequencing data [3]. It also can be configured to use new releases of the IMGT database as they become available.

If an allele candidate has not been determined by the end of the run, HLA-HD will output multiple candidates. MHC Hammer will randomly choose an allele pair from the equally likely candidates, but give priority to those that have a full sequence available in the IMGT database. If only a partial sequence is available, the missing section of the genomic sequence is replaced with the corresponding section from the most similar allele that does have a full sequence.

Constructing the patient-specific HLA references

A set of reference files are created for each individual, containing the sequence information of the individual's class I alleles. These reference files include:

- A genomic reference, which contains the complete allele sequence
- A transcriptomic reference, which contains the exon allele sequence

Alignment steps

Three sets of alignments are created with MHC Hammer: NovoAlign (v3.09.4, http://novocraft.com/) is used with both the WES and RNA-seq data, and STAR[4] (v2.7.10a) is used with the RNA-seq data.

Aligning the WES and RNA-seq data using NovoAlign

The reads in the HLA FASTQ file are mapped to the HLA reference using NovoAlign with parameters that allow reads to map to multiple locations (-R 0 -r All 9999 -o SAM -o FullNW). The genomic reference is used if the input FASTQ files are from WES and the transcriptomic reference is used if the input FASTQ files are from RNA-seq.

Aligning the RNA-seq data using STAR

The RNA-seq reads in the HLA FASTQ file are also mapped to the HLA genomic reference using the STAR aligner, which can account for gaps in the alignment resulting from introns or alternative splicing.

When mapping to a small region of the genome, such as the 6 class I HLA alleles, STAR will spend a large amount of time trying to find poor quality alignments for reads that originate outside the small region. Therefore, to reduce the computational time required by STAR, the RNA-seq reads in the HLA FASTQ are further filtered to only include those that contain a 30-base pair sequence from the IMGT database.

A two-pass alignment strategy is used to improve accuracy of novel splice junction detection [5]. This involves:

1. Aligning the HLA reads to the patient specific reference using STAR. This results in a table of splice junctions for each sample. In this run, the STAR parameters changed from default are:
    a. outFilterMultimapNmax=20
    b. alignSJoverhangMin=3
2. The splice junction tables across all the samples are concatenated and filtered such that a splice junction must pass the following thresholds in at least one sample to make it into the final concatenated table:
    a. intron motif (column 5) > 0 (i.e. must be a canonical splice junction)
    b. At least 2 uniquely mapping reads supporting the splice junction
    c. Must be an unannotated splice junction (column 6 = 0)
3. On a per sample basis we add to the filtered HLA reads any read in the input BAM file that contains the 14-base pair sequence around any novel splice junction detected in the first STAR alignment
4. These reads are then realigned with STAR to the patient-specific reference to get, for each sample, a STAR BAM file and a table of splice junctions. In this run the following STAR parameters are changed from the default:
    a. outFilterMultimapNmax=10
    b. outFilterMismatchNmax=1
    c. alignSJoverhangMin=3
    d. alignSJDBoverhangMin=1
    e. sjdbScore=1

Filtering the HLA allele BAM files

The WES and RNA-seq HLA BAM files from NovoAlign and STAR are then filtered such that reads with more than one mismatch to the HLA reference are removed. The number of mismatches allowed can be adjusted using the parameter `max_mismatch`.

Calculating HLA SNP positions

For each HLA gene, the HLA allele sequences are aligned to identify the positions at which the sequences differ, called the SNP positions.

DNA analysis

Calculating the DNA HLA allelic copy number

The DNA HLA allelic copy number is calculated using the B-Allele Frequency (BAF) and logR at the SNP positions. These SNP positions are filtered to only include SNPs with depth higher than the input parameter `min_depth` in the germline HLA BAM file. For each filtered SNP_i, the BAF is calculated as

$$BAF_i = \frac{allele\ 1\ tumour\ depth\ at\ SNP\ i}{total\ depth\ at\ SNP\ i}$$

where the choice of allele 1 is arbitrary.

The logR is calculated at each position in the allele that has a depth higher than the input parameter `min_depth`, as

$$logR = log2(\frac{tumour\ depth}{germline\ depth} \times \frac{germline\ library\ size}{tumour\ library\ size})$$

The allele is then split into 150-base pair bins and the median logR is calculated for each bin.

The copy number of each filtered SNP in allele 1 and allele 2 is then calculated as

$$allele1\ CN_i = \frac{\rho-1+BAF_i\times 2^{logR_i}\times(2(1-\rho)+\rho\varphi)}{\rho}$$

$$allele2\ CN_i = \frac{\rho-1-2(BAF_i-1)^{logR_i}\times(2(1-\rho)+\rho\varphi)}{\rho}$$

where ρ is the purity of the tumour region, φ the ploidy of the tumour region and $logR_i$ is the median logR of the bin that SNP_i falls in. The allele copy number is the median copy number across the SNPs.

Calculating the DNA HLA allelic imbalance

To measure DNA allelic imbalance (AIB) in the HLA genes, we use the logR at the SNP positions. These SNP positions are filtered to only include SNPs with depth higher than the input parameter `min_depth` in the germline HLA BAM file. Before calculating the logR, the coverage at the SNP positions is first adjusted so that each sequencing read is only counted once per SNP. To do this, a read that overlaps more than one SNP is randomly assigned to

count towards one of the SNPs it overlaps. A paired Wilcoxon test with a significance threshold of $p<0.01$ is used to determine if there is a significant difference in the logR at the filtered SNP positions between the two alleles.

Evaluating loss of heterozygosity

For a given class I HLA gene, HLA LOH is called if both of the following are true:

- The copy number of the minor allele is less than 0.5
- There is DNA AIB

Calling HLA allelic somatic mutations

To call somatic HLA allelic mutations, Mutect2[6] (v4.1.8.1) is run on the germline/tumour pairs of WES HLA BAM files. The mutation calls are filtered using the GATK[7] function FilterMutectCalls (v4.1.8.1). The Ensembl Variant Effect Predictor[8] (VEP, v109.3) is used to predict mutation consequence.

Calculating the DNA expected depth

The expected depth (ED) estimates the depth of the reads that are coming solely from the cancer cells. This is calculated from the depth of the matched germline sample and the purity of the tumour region. For a given allele, the expected depth of SNP_i is:

$$ED_i = \quad depth\ of\ SNP_i\ in\ the\ germline\ \times\ \rho \times \frac{tumour\ library\ size}{normal\ library\ size}$$

where ρ is the purity of the tumour region. The expected depth of the allele is the median of the expected depth at the filtered SNP positions. If the expected depth is too low, we would not expect to have the required coverage to accurately classify LOH, even if it were present.

RNA analysis

Quantifying HLA allelic expression

Some individuals may carry two very similar or identical (homozygous) alleles for a given HLA gene. In these cases, sequencing reads may map equally well to both alleles for that gene. In addition, it is possible that two alleles from different genes share similar sequences, and in this case reads may map equally well to the two alleles from different genes. If these multi-mapping reads are not accounted for when calculating HLA expression, the estimates of HLA allele expression may be inflated.

To quantify HLA allelic expression, an updated read count for each allele that accounts for multimapping reads is first calculated. To do this, the fraction of reads that map uniquely to allele 1 (f_1) is defined as:

$$f_1 = \frac{r_1}{r_1 + r_2}$$

where r_1 is the number of reads that map uniquely to allele 1 and r_2 is the number of reads that map uniquely to allele 2 and the choice of allele 1 is arbitrary.

Using f_1, an updated read count for each allele is calculated as:

$$R_1 = r_1 + f_1 \times r_{12}$$
$$R_2 = r_2 + (1 - f_1) \times r_{12}$$

where r_{12} is the number of reads mapping to both alleles of a given gene, R_1 is the updated read count for allele 1, and R_2 is the updated read count for allele 2.

Using this updated read count, the allele expression, with units reads per kilobase million (RPKM), is calculated for allele 1 ($RPKM_1$) and allele 2 ($RPKM_2$) as:

$$RPKM_1 = \frac{R_1/l}{a_1}$$
$$RPKM_2 = \frac{R_2/l}{a_2}$$

where l is the library size divided by a factor of one million, a_1 and a_2 are the lengths of allele 1 and allele 2 in kilobase units, respectively.

The gene level expression ($RPKM$) is calculated as:

$$RPKM \ = RPKM_1 \ + \ RPKM_2$$

Calculating RNA allelic imbalance

To measure RNA AIB in the HLA genes, we use the read depth at the SNP positions. The read depth is adjusted so that each sequencing read is only counted once per SNP. To do this, a read that overlaps more than one SNP is randomly assigned to count towards one of the SNPs it overlaps. A paired Wilcoxon test per gene with a significance threshold of $p<0.01$ is used to determine if there is a significant difference in the read depth at the SNP positions.

Calculating HLA allelic repression

We define a given HLA allele as being repressed if it has significantly lower expression in the tumour sample compared to the normal sample.

To calculate this, for a given allele, the read depth at the SNP positions are normalised to account for differences in the library size between the two samples by multiplying the normal read depth by the tumour library size divided by the normal library size.

A given allele is called as repressed in the tumour compared to the normal if the following are true:

1. There is a significant difference in the normalised read depths between the tumour and the normal sample. This is determined using a paired Wilcoxon test with a significance threshold of $p<0.01$.
2. The median normalised depth at the SNP positions in the tumour sample is lower than the median normalised depth at the SNP positions in the normal sample.

Calling HLA alternative splicing

Detecting complete exon skipping, partial exon skipping and partial intron retention

To detect complete exon skipping, partial exon skipping and partial intron retention in the HLA alleles we use the novel splice junctions identified by STAR in the two-pass alignment. A splice junction is the position at which two spliced fragments of mature mRNA join after splicing and can be represented as two genomic coordinates that indicate the start (S^s) and end (S^e) of the sequence of nucleotides that is spliced out of the mRNA transcript. Each splice junction can be classified as “known”, meaning that it represents an intron described in the sequence information in the IMGT database, or “novel”, meaning that it is not described in the IMGT database. Each novel splice junction is classified as either a complete exon skipping event, a partial exon skipping event or a partial intron retention event using the definitions outlined in Figure 1.

Detecting complete intron retention

A complete intron retention event will not cause a novel splice junction as no nucleotides will be spliced out. However, if an intron is retained in the mature mRNA, it will increase the number of RNA-seq reads aligning to that intron. Therefore, to investigate the presence of complete intron retention, MHC Hammer calculates the coverage of the exons and introns across all the alleles using the bioinformatic tool mosdepth[9]. The depth is normalised by dividing by the length of the exon or intron.

Calculating the novel transcript proportion

To estimate the relative abundance of the novel (alternatively spliced) transcripts compared to the canonical (non-alternatively spliced) transcripts (the novel transcript proportion), MHC Hammer calculates the number of uniquely mapping reads containing the novel splice junction (yellow in Figure 1) divided by the total number of reads that could contain the splice junction. The total number of reads that could contain the splice junction is calculated as the sum of the number of uniquely mapping reads containing the novel splice junction (yellow in Figure 1), and the number of reads that contain the corresponding known splice junction (green in Figure 1).

Calculating the purity-scaled novel transcript proportion

To estimate the fraction of cancer cells that harboured an alternative splicing event, a purity-scaled novel transcript proportion is calculated. To do this, the novel transcript proportion

is divided by the purity to account for the non-cancer cells in the tumour region. As the purity-scaled proportion represents the fraction of cancer cells that carry the somatic alternative splicing event, the purity-scaled proportion is capped at 1.

Consequences of the HLA alternative splicing events

An alternative splicing event will change the mRNA sequence of the allele and in doing so may introduce a frameshift or a premature termination codon.

To determine the consequence of an alternative splicing event, the new sequence that results from the novel splice junction is calculated. For complete or partial exon skipping, the new sequence is calculated by removing the coding sequence that falls between the start (S^s) and end (S^e) of the novel splice junction. For events where the start of an intron is retained, the new sequence is calculated by inserting the sequence from the start of the intron (i_i^s) to the start of the novel splice site (S^s). For events where the end of an intron is retained, the new sequence is calculated by inserting the sequence from the end of the novel splice junction (S^e) to the end of the intron (i_i^e).

Once the new sequence has been determined, the alternative splicing event is defined as inframe if the number of bases in the new sequence is divisible by 3 and a framshift event otherwise. The alternative splicing event is defined as introducing a premature termination codon if a stop codon has been introduced into the new sequence.

Tumour-enriched and tumour-depleted HLA alternative splicing

We use a Fisher's exact test with a significance threshold of $p<0.01$ to determine if a HLA alternative splicing event is enriched in the tumour (tumour-enriched), depleted in the tumour (tumour-depleted), or neither. Specifically, we test whether there is an enrichment in the number of reads that do/do not support the alternative splicing event in the tumour compared to the number of reads that do/do not support the alternative splicing event in the normal.

Tumour-to-normal change in the novel transcript proportion

If the novel splice junction proportion in the tumour is greater than the novel splice junction proportion in the normal, the tumour-to-normal change in the novel splice junction proportion is defined as:

$$\frac{splice\ junction\ proportion\ in\ tumour - splice\ junction\ proportion\ in\ normal}{splice\ junction\ proportion\ in\ tumour}$$

If the novel splice junction proportion in the tumour is less than or equal to the novel splice junction proportion in the normal, the tumour-to-normal change in the novel splice junction proportion is defined as:

$$-1 * \frac{splice\ junction\ proportion\ in\ normal - splice\ junction\ proportion\ in\ tumour}{splice\ junction\ proportion\ in\ normal}$$

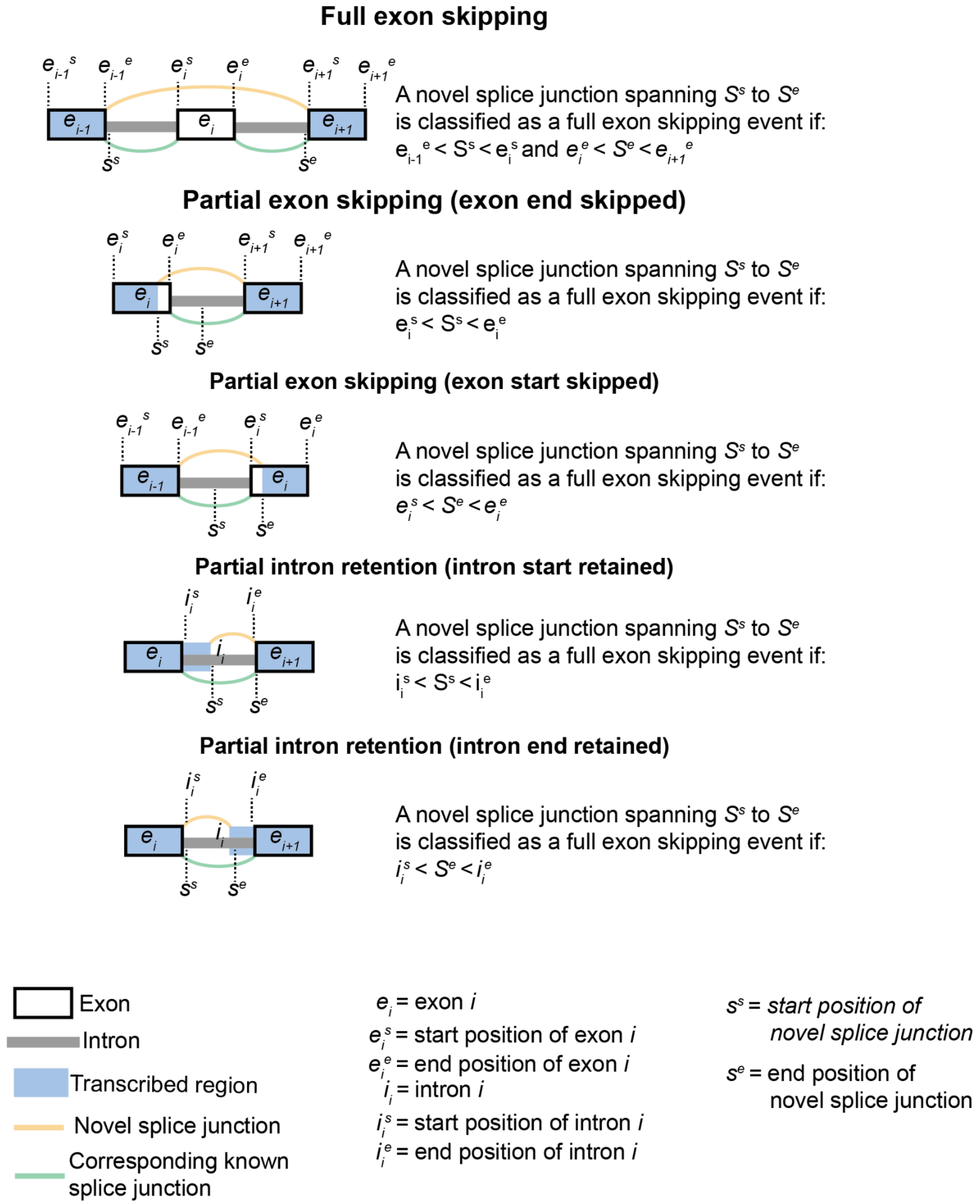

Figure 1 | The classification of a novel splice junction with start site S^s and end site S^e.

References

1. Lefranc, M.-P. *et al.* IMGT®, the international ImMunoGeneTics information system® 25 years on. *Nucleic Acids Res.* **43**, D413–D422 (2014).
2. Kawaguchi, S., Higasa, K., Shimizu, M., Yamada, R. & Matsuda, F. HLA-HD: An accurate HLA typing algorithm for next-generation sequencing data. *Hum. Mutat.* **38**, 788–797 (2017).
3. Liu, P. *et al.* Benchmarking the Human Leukocyte Antigen Typing Performance of Three Assays and Seven Next-Generation Sequencing-Based Algorithms. *Front. Immunol.* **12**, 652258 (2021).
4. Dobin, A. *et al.* STAR: ultrafast universal RNA-seq aligner. *Bioinformatics* **29**, 15–21 (2013).
5. Veeneman, B. A., Shukla, S., Dhanasekaran, S. M., Chinnaiyan, A. M. & Nesvizhskii, A. I. Two-pass alignment improves novel splice junction quantification. *Bioinformatics* **32**, 43–49 (2016).
6. Benjamin, D. *et al.* Calling Somatic SNVs and Indels with Mutect2. *bioRxiv* 861054 (2019) doi:10.1101/861054.
7. McKenna, A. *et al.* The Genome Analysis Toolkit: a MapReduce framework for analyzing next-generation DNA sequencing data. *Genome Res.* **20**, 1297–1303 (2010).
8. McLaren, W. *et al.* The Ensembl Variant Effect Predictor. *Genome Biol.* **17**, 122 (2016).
9. Pedersen, B. S. & Quinlan, A. R. Mosdepth: quick coverage calculation for genomes and exomes. *Bioinformatics* **34**, 867–868 (2018).

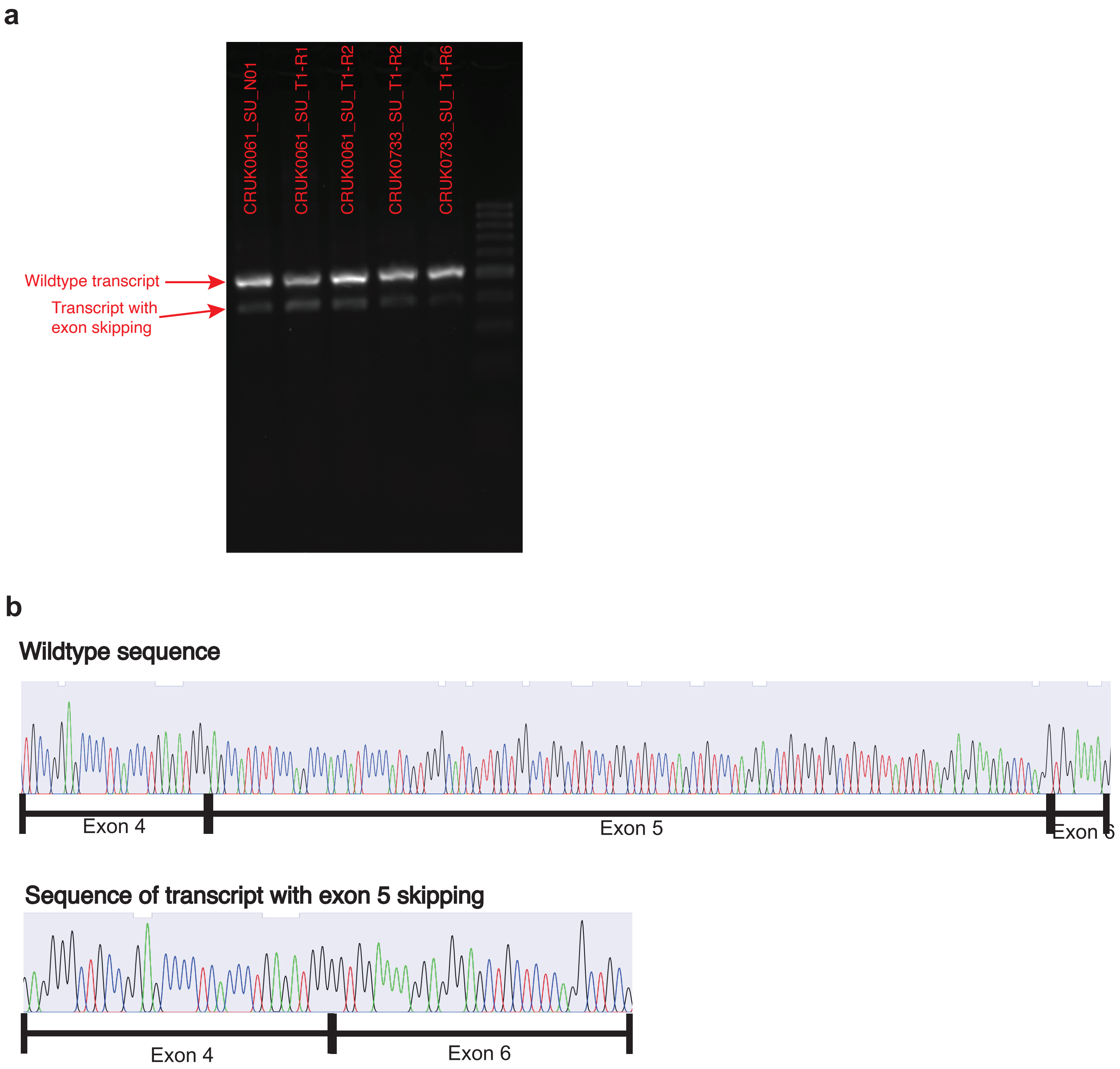

Supplementary Figure 1 | **a** PCR product highlighting the different lengths of the wildtype transcript and transcript with exon 5 skipping. **b** The sequence of the wildtype transcript and transcript with exon 5 skipping, determined from Sanger sequencing. Each peak represents a single nucleotide; A is green, T is red, C is blue and G is black.

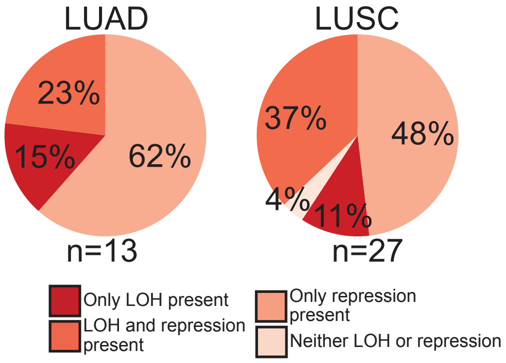

Supplementary Figure 2 | The rate of HLA loss of heterozygosity (LOH) and repression in the TCGA lung adenocarcinoma (LUAD) and lung squamous cell carcinoma (LUSC) cohorts.

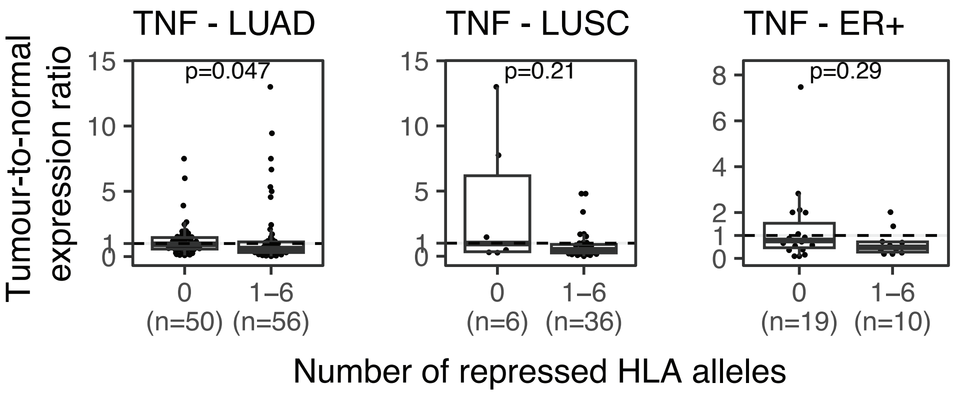

Supplementary Figure 3 | The relationship between the tumor-to-normal ratio of TNF alpha (TNF) expression and the number of transcriptionally repressed alleles in the tumor region. P values were derived from a two-sided Wilcoxon test. Boxplots show median and first and third quartiles, and whiskers extend up to 1.5 × IQR above and below the IQR. LUAD: lung adenocarcinoma; LUSC: lung squamous cell carcinoma; ER+: estrogen receptor positive.

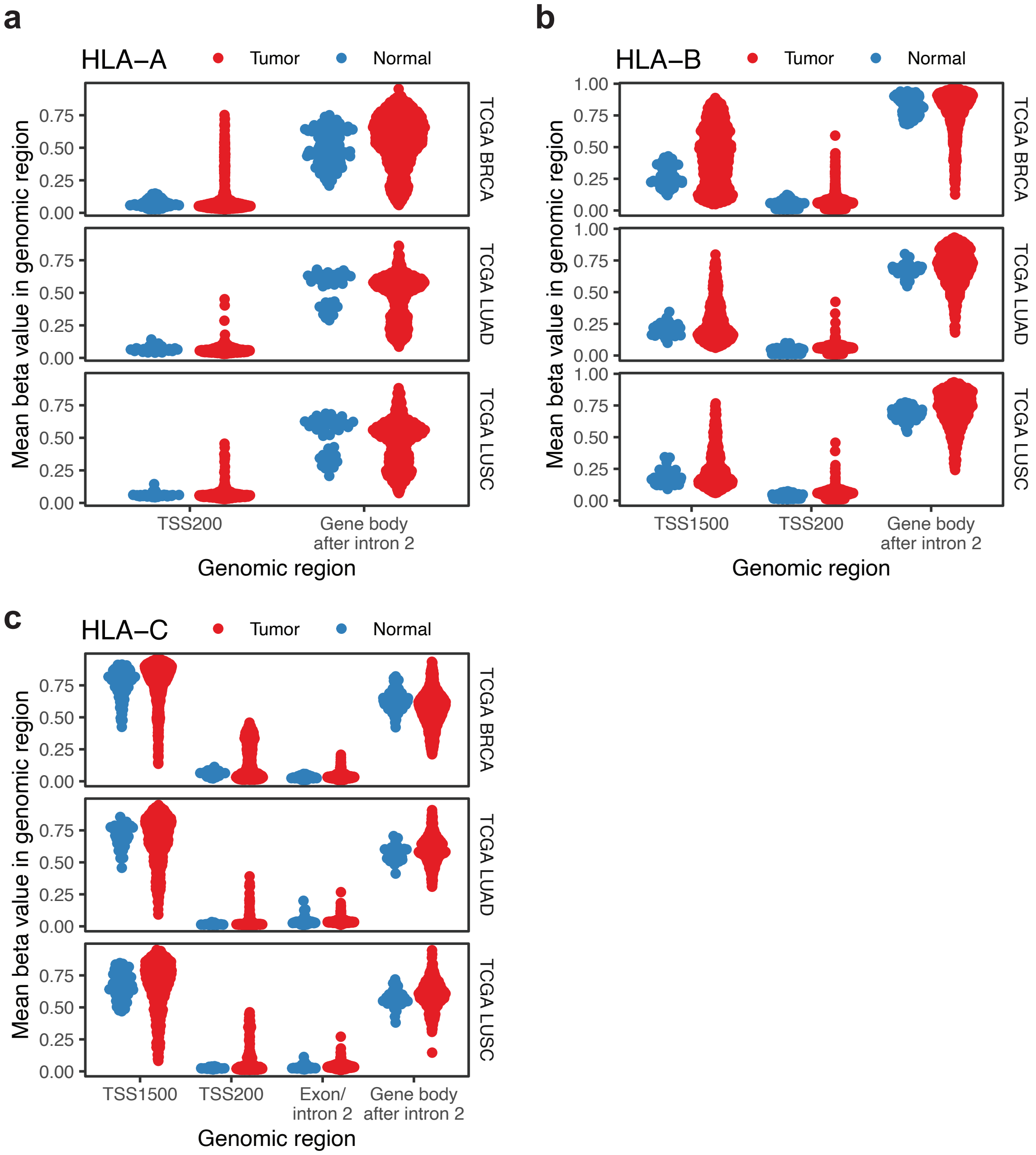

Supplementary Figure 4 | For each TCGA sample, the average methylation beta value in each of the four regions of the HLA genes is shown. BRCA: breast cancer, LUAD: lung adenocarcinoma, LUSC: lung squamous cell carcinoma, TSS1500: the region 1500-200 bp upstream of the transcription start site, TSS200: the region 200 bp upstream of the transcription start site.

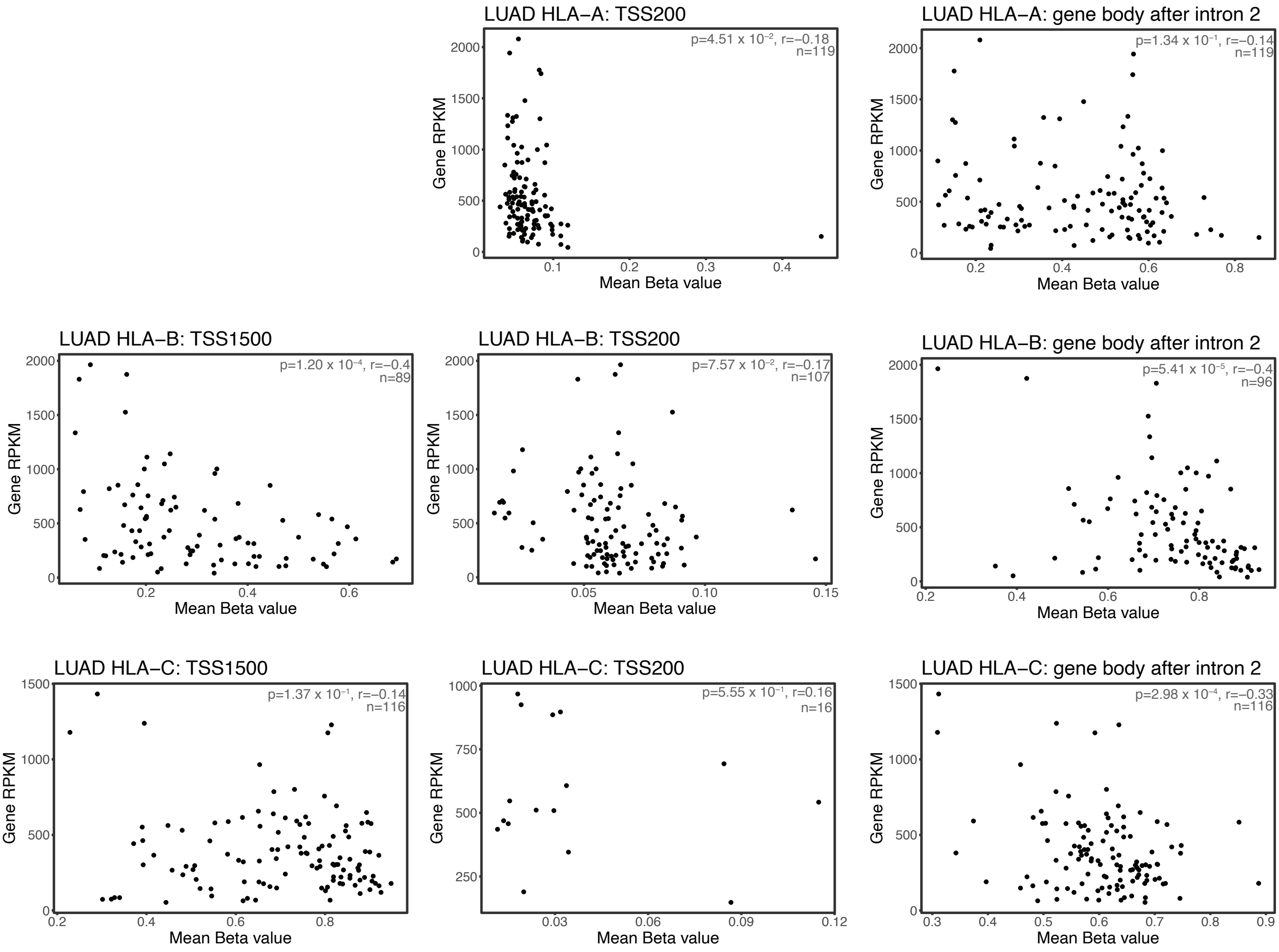

Supplementary Figure 5 | The correlation between the average methylation beta value and the expression of the HLA gene in the TCGA LUAD tumor samples. Correlation coefficients (r) and corresponding P values were calculated with the Pearson's correlation method. LUAD: lung adenocarcinoma; TSS1500: the region 1500-200 bp upstream of the transcription start site; TSS200: the region 200 bp upstream of the transcription start site; RPKM: reads per kilobase per million.

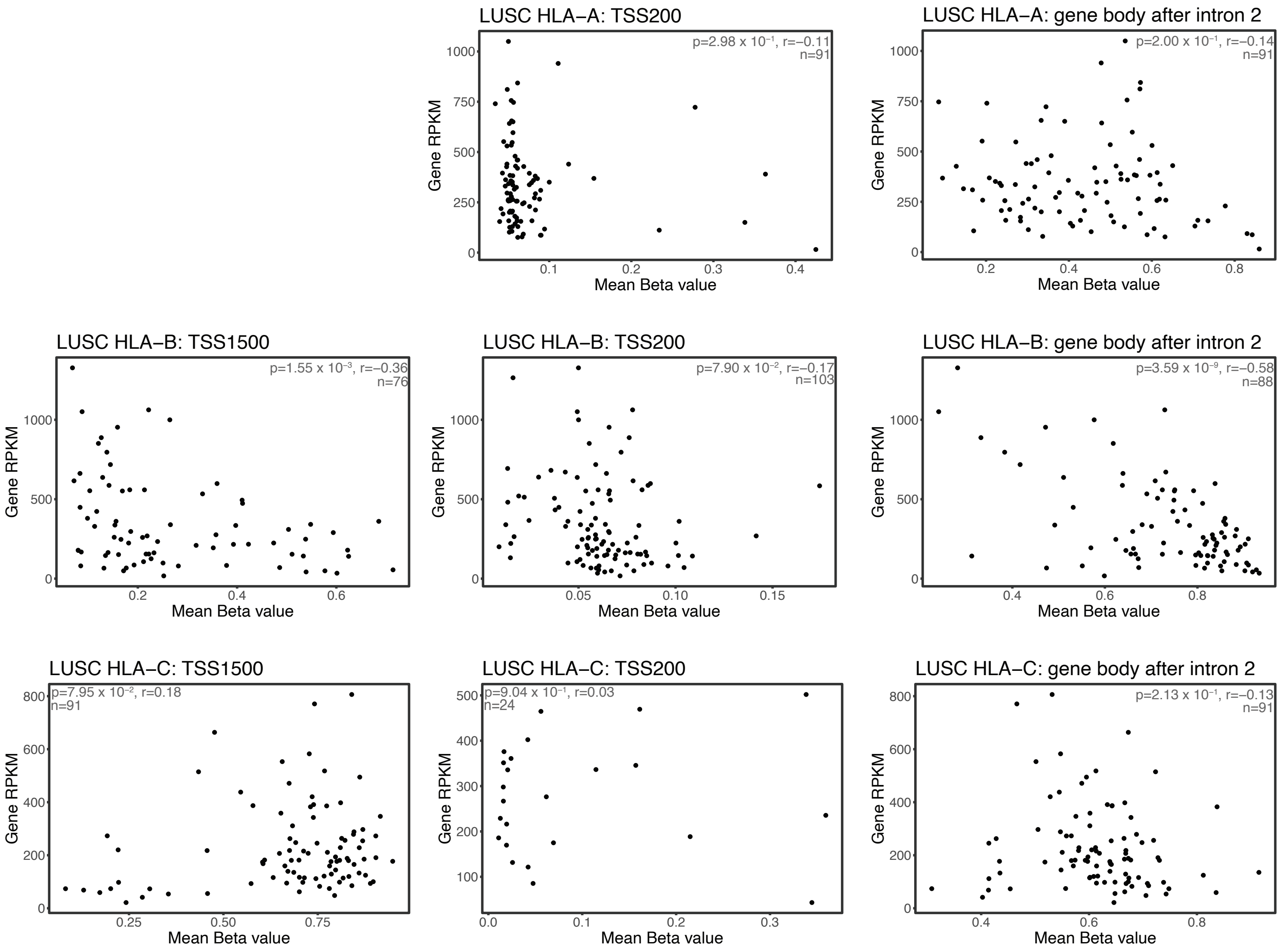

Supplementary Figure 6 | The correlation between the average methylation beta value and the expression of the HLA gene in the TCGA LUSC tumor samples. Correlation coefficients (r) and corresponding P values were calculated with the Pearson's correlation method. LUSC: lung squamous cell carcinoma; TSS1500: the region 1500-200 bp upstream of the transcription start site; TSS200: the region 200 bp upstream of the transcription start site; RPKM: reads per kilobase per million.

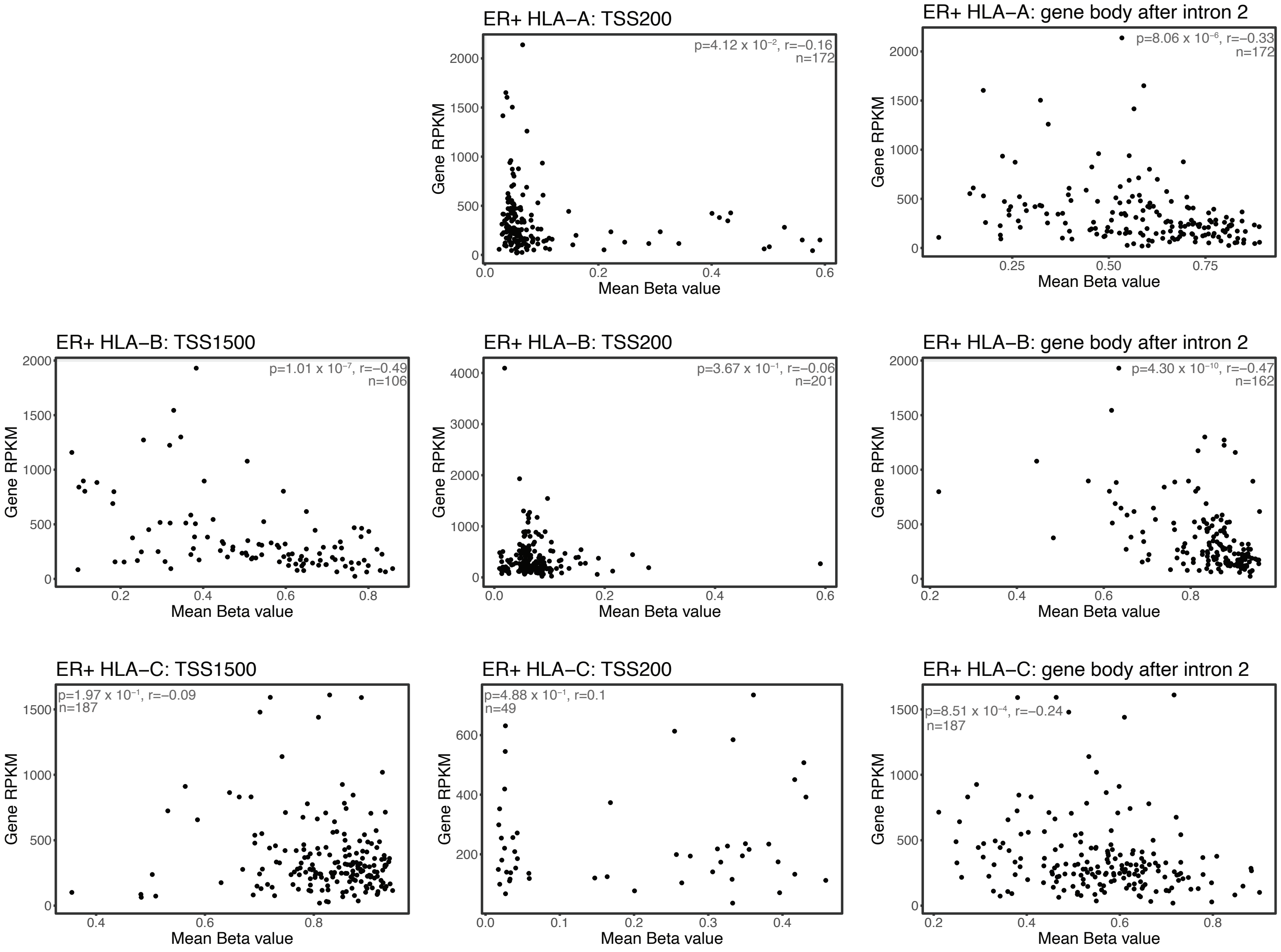

Supplementary Figure 7 | The correlation between the average methylation beta value and the expression of the HLA gene in the TCGA ER+ tumor samples. Correlation coefficients (r) and corresponding P values were calculated with the Pearson's correlation method. ER+: estrogen receptor positive; TSS1500: the region 1500-200 bp upstream of the transcription start site; TSS200: the region 200 bp upstream of the transcription start site; RPKM: reads per kilobase per million.

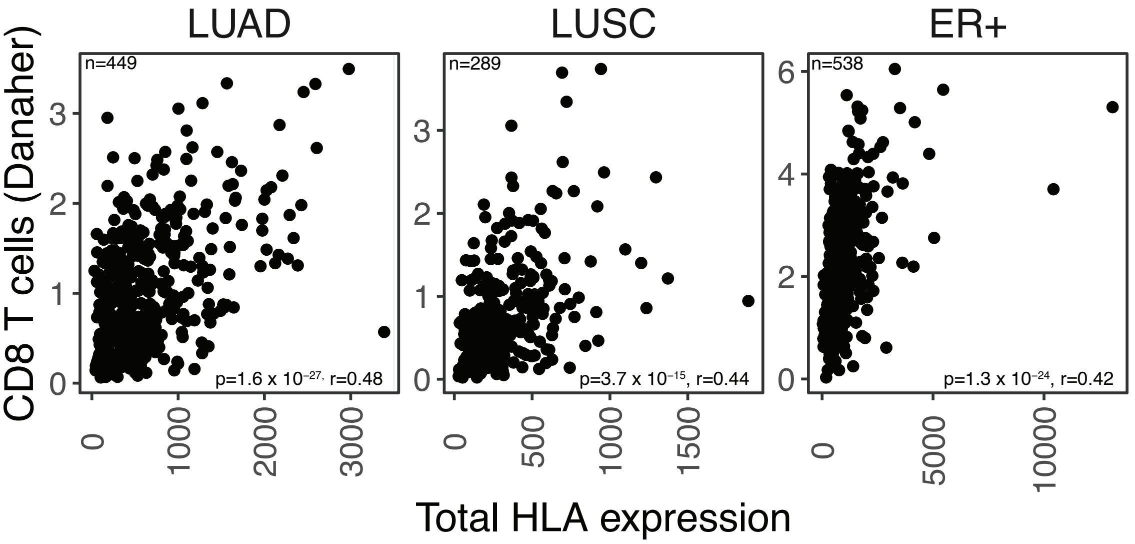

Supplementary Figure 8 | The relationship between total HLA expression and the amount of CD8 T cell infiltration. Correlation coefficients (r) and corresponding P values were calculated with the Pearson's correlation method. LUAD: lung adenocarcinoma; LUSC: lung squamous cell carcinoma; ER+: estrogen receptor positive.
